# In Situ 3D Crystal Growth Measurement: Unravelling the Nature of Crystal Face-Specific Growth Rate Dispersion in α‑Form l‑Glutamic Acid

**DOI:** 10.1021/acs.iecr.6c02035

**Published:** 2026-09-30

**Authors:** Chen Jiang, Thomas P. Ilett, Thomas A. Hazlehurst, Cai Y. Ma, David C. Hogg, Kevin J. Roberts

**Affiliations:** † School of Chemical and Process Engineering, 4468 University of Leeds, Leeds LS2 9JT, U.K.; ‡ School of Computer Science, 4468 University of Leeds, Leeds LS2 9JT, U.K.

## Abstract

Obtaining 3D crystal morphology and its evolution during growth is beneficial for crystallization process design. A newly developed image analysis tool is used to reconstruct the complex 3D morphology (20 habit faces in 6 forms) of α form l-glutamic acid crystals from 2D transmission microscopy images captured in situ during the growth process. A digital crystallographically based polyhedral mesh is fitted to experimental images, enabling calculation of face-specific growth rates. Solution supersaturation during the growth process is estimated using the fitted crystal volume together with solubility and density data. The data reveals significant growth rate dispersion, reflecting differences in the local solution environment, with {110} and {100} faces growing fastest and {001} the slowest. For our experiments, mechanistic analysis is consistent with surface integration-limited growth, which aligns with the BCF model, except for the {111} and {001} surfaces, which are mass transfer limited under the B&S model.

## Graphical abstract

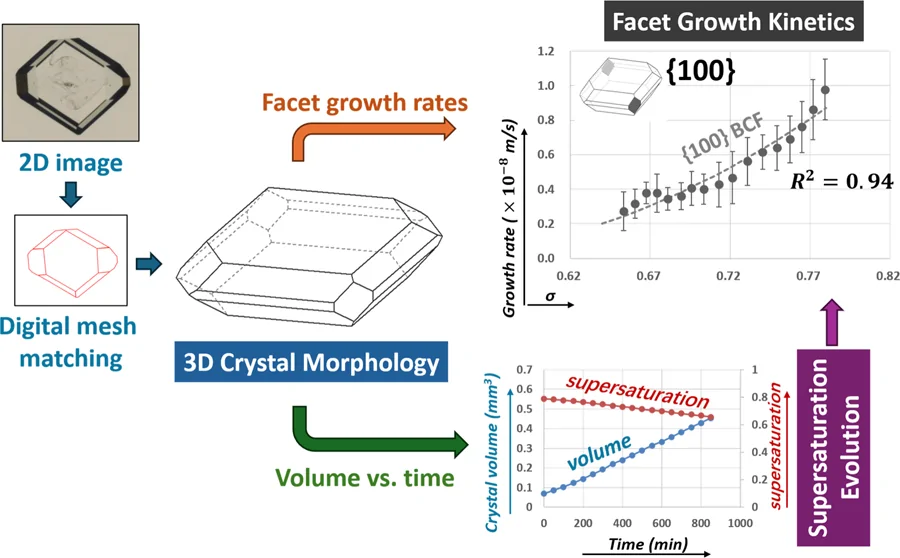

## 1 Introduction

Industrial crystallization from the solution phase is important in the manufacture of solid-form fine chemical products such as food, pharmaceuticals and agrochemicals. The size and shape of crystal products are critical factors influencing downstream processes such as filtration, storage, and transportation.^1^ Variation in crystal polymorphic form may also further impact the final performance or efficiency of the product, such as stability, dissolution. Crystal size and shape evolution during crystallization processes can be significantly affected by any variation in crystal facet growth kinetics and hence the accurate determination of the latter can be crucial for the prediction and control of crystal morphology.^2–5^ This is an important aspect of crystallization process design and has been extensively studied for many years.^1^

Crystal growth kinetics and mechanisms have been studied based upon the well-known interfacial growth models such as Burton–Cabrera–Frank (BCF),^6^ Birth and Spread (B&S),^7^ and the power law model.^8,9^ In practice, the crystal growth rate is also affected by the rate of solute diffusing from the bulk solution to the individual crystal habit surfaces, referred to as the solute mass transfer (MT) process,^10^ as can be demonstrated by comparing the growth behavior of crystals from stagnant and stirred solution.^5,11–13^ Growth kinetic models incorporating the effects of solute MT upon the overall mechanisms of crystal growth using interface-specific functions to fit the growth rate data have been demonstrated for the case of methyl stearate by Camacho et al.^14^ Through this approach, the influence of the diffusion of solute growth units in the bulk solution due to solute MT and their incorporation onto the crystal surface (surface integration, SI) can be numerically assessed and quantified in order to determine their relative magnitudes, hence determining which of these two steps is rate-limiting with respect to the overall process.

Online imaging techniques have been performed to monitor the real-time growth of crystals through many crystallization process studies, including the usage of optical microscopy with the camera located either inside or outside of a reactor in order to investigate the growth and development of a population of crystals.^15–19^ However, due to crystallization reactor agitation and the resultant motion of the population of crystals inside the reactor, it can be very difficult to track the growth behavior of an individual single crystal within a representative crystallization reactor environment throughout its entire growth process. More precise studies have hence used temperature-controlled ‘growth cell’ systems in order to focus on monitoring the growth of a single crystal under well-controlled conditions.^20^ This approach can provide images of one crystal with very high quality, enabling accurate growth measurements of the individual crystal facets. Such growth cell systems have also been used for studying growth rates of various compounds, such as ibuprofen,^12,21^ para-aminobenzoic acid,^22^ and tolfenamic acid.^23^

Crystal growth rate dispersion (GRD) has been described by Janse and de Jong^24^ as a phenomenon whereby different crystals within the population grow at different rates under apparently the same growth conditions. Since then, GRD has been widely observed and investigated for various compounds, albeit through studies mostly in batch crystallization processes such as potassium alum,^25^ d-fructose,^26^ ammonium dihydrogen phosphate,^27^ and α-lactose monohydrate.^28^ GRD has also been demonstrated to have a significant effect on particle size distribution,^4,24^ and has also been observed on the different crystallographically independent habit faces of potassium alum crystals,^29^ demonstrating its impact on the morphological evolution of the crystal. Attempts to physically model GRD from a mechanistic standpoint have been made by van der Heijden,^30,31^ and these have also highlighted that the presence of lattice strain and defects within the crystals may also affect the growth process and hence mediate their growth rate behavior.

The effect of dislocations and strain on GRD has also been experimentally assessed by Sherwood,^32^ revealing that both edge and screw dislocation defects can potentially influence the growth process and that the rate of growth increases with higher growth dislocation density.^33^ Interestingly, these studies also highlight that variation in growth rate can also depend on the degree of lattice strain present within the crystals, with the more strained crystals tending to grow at lower rates compared to the unstrained cases.^34^ The origins of GRD can be quite complex when mediated by the presence and influence of lattice strain and dislocations, with the former being regarded as a major potential influence on the anomalous low growth rates observed for smaller-sized crystals.^32,35^ The importance of lattice defects on crystal growth rates and mechanisms is also very well-known,^1,6^ notably through screw dislocations emerging on crystal facet surfaces, creating permanent step sites, which can greatly facilitate surface nucleation and integration.

It is an assumption that the growth of crystallographically equivalent faces within the overall 3D crystal morphology of a given form should be quite similar. However, this lacks detailed verification and probably ignores any variations in lattice defect structure between the crystallographically ‘equivalent’ habit faces as well as the impact of any differences in the solute MT from the solution to the growth interface^36,37^ within the local crystallization environment.

l-Glutamic acid (LGA) is an amino acid widely used in the food and pharmaceutical industries and one that has also been widely used as a representative compound in crystallization process research.^38^ It is well known that LGA has two monotropically related conformational polymorphs: the metastable prismatic-shaped α form and the stable needle-shaped β form.^39,40^ Both polymorphs crystallize in an orthorhombic structure in the *P*2_1_2_1_2_1_ space group.^41^ In previous studies, the crystals of both polymorphs and their morphologies have been observed and characterized.^40,42,43^

The theoretical growth morphologies for both polymorphs when crystallized from aqueous solutions have been predicted by Turner et al.,^41,44^ with the crystals for both polymorphic forms matching reasonably well with crystals observed experimentally. Habit surfaces that exhibit more favorable solvent interactions tend to have reduced growth rates due to the retarding impact of desolvation at the crystal/solution interface during growth and thiscan lead, in turn, to an increase in facet surface area within the final morphology.^41^

Under certain conditions, the α form LGA crystals (α-LGA) can undergo a solvent-mediated transformation into the β form crystals (β-LGA). This transformation process was first observed and studied by Kitamura,^40^ who also found the transformation rate seems to increase with higher temperature. Since then, the transformation process has attracted much academic attention.^16,45–49^ Previous studies have also been monitoring this transformation process with various approaches, such as imaging techniques^16,17^ or online X-ray diffraction.^46,47^ The transformation process may also be affected by the presence of different surfactants,^49^ and it has been observed that the α form crystals can actually provide templating surfaces for the secondary nucleation and subsequent growth of the β form crystals.^45,48^ Moreover, the overall growth process has been observed to be affected by the presence of additives such as l-phenylalanine.^42^

Previous studies have investigated the growth rate of β-LGA crystals using process analytical techniques such as ATR-FTIR spectroscopy^2^ and FBRM or imaging techniques,^50^ but these measurements tend to be restricted to 1D (particle equivalent diameter or chord length) or 2D (length and width) dimensions due to the limitations of instrumentation and suitable image capture and analysis issues. Using the knowledge of the fixed angles between the constituent habit faces in a single crystal, the facet growth rates of the β-LGA crystal have been indirectly calculated from their projected lengths.^42,43^ Recently, with the development of improved image processing techniques, facet-specific growth rates of β-LGA have been measured directly from images with the aid of AI-based image analysis tools.^38^ Using the shadow of the crystal under transmitted light, it has also been possible to estimate the height of the crystal^51^ during the growth process and this, combined with the lateral (2D) measurements, has enabled characterization of growth rates in 3D for this case. However, this method is only applicable for β-LGA crystals because of its simple needle-like morphology.

The α form has a more complex crystal morphology when compared to the β form. The growth of α-LGA has also been measured in the length and width directions using imaging techniques^42,43^ or process analytical techniques.^52–54^ Similarly tothe β form, the facet growth rates for the {111} and {001} faces of α-LGA have been estimated from the lateral growth rates on the length direction in combination with the known angle between the appropriate crystal habit faces.^42,43,55^ However, compared to the comparatively simple crystal habit of the β form crystals, it is a much more complicated problem for directly obtaining facet growth rates for the α form crystals, not only because the growth of the metastable α form is more difficult to control than for the β form, but also due to the complex array of different habit faces within its prismatic morphological structure, which is characterized by many tilted faces with respect to the axis of observation. To our knowledge, there have been no direct measurements of the growth rates of all the individual crystal surfaces of α-LGA or any other faceted crystals reported in literature. Furthermore, there has been no reliable method for measuring the 3D growth rate for individual crystal habit faces. Existing approaches have mostly provided average particle size estimated from volume-equivalent diameter.^56^

In this study, the growth rates from the solution phase for all the crystal surfaces of α-LGA have been measured in situ using a crystal growth cell together with transmitted light optical microscopy. For the case of a single α-LGA crystal, a newly developed 3D shape fitting method^57^ has been applied here in order to reconstruct its full 3D morphology initially, and then its temporal development during the growth process directly from the projected 2D images as observed by transmission optical microscopy. The aim of the work presented here is to assess whether accurate growth rate measurements can be achieved using this new approach and then apply these to the simultaneous characterization of all the habit faces present in an α-LGA single crystal and, through this, characterize the growth kinetics for these faces. In doing so, any variations in the growth behavior between crystal surfaces that are both crystallographically equivalent and non-equivalent can also be assessed.

## 2 Materials and Methods

### 2.1 Materials

l-Glutamic acid (LGA, C_5_H_9_NO_4_, molecular weight: 147.13 g/mol) was used in this study. Dry β-form LGA was purchased from Sigma-Aldrich with a purity of ≥99%. LGA solutions were made up using deionized water.

The crystal structure for the α form was obtained from the Cambridge Crystallographic Data Centre (CCDC)^58^ structural database (ref code LGLUAC02 ^59^), revealing that the molecules were packed in a tetramolecular orthorhombic structure in space group *P*2_1_2_1_2_1_ with unit cell parameters: *a* = 7.068 Å, *b* = 10.277 Å, and *c* = 8.755 Å.

### 2.2 Experimental Setup for Growth Rate Studies

A crystal growth cell system,^38^ as shown in Figure 1, was used in this study. The system includes a detachable 0.5 mL glass cuvette (54 × 10 × 1 mm) fixed within a sealed growth cell connected to a HUBER Ministat 125 recirculating water bath fitted with a Pilot ONE controller to achieve accurate and stable temperature control (±0.02 °C stability with respect to the temperature set point). Both the cuvette and the growth cell (top and bottom) windows were built of high-quality optical glass, allowing an optimal condition for in situ monitoring using transmission mode digital optical microscopy to enable the continuous capture of images at regular intervals of the crystals growing within the cuvette. A Keyence VHX7000 digital microscope^60^ was used for imaging, whose facilities encompass 3 zooming lenses (20–100×, 100–500×, 500–2500×), a 1/1.7-in. 4K CMOS image sensor (108 megapixels) camera, all under software control from a controlling PC.

**Figure 1:**
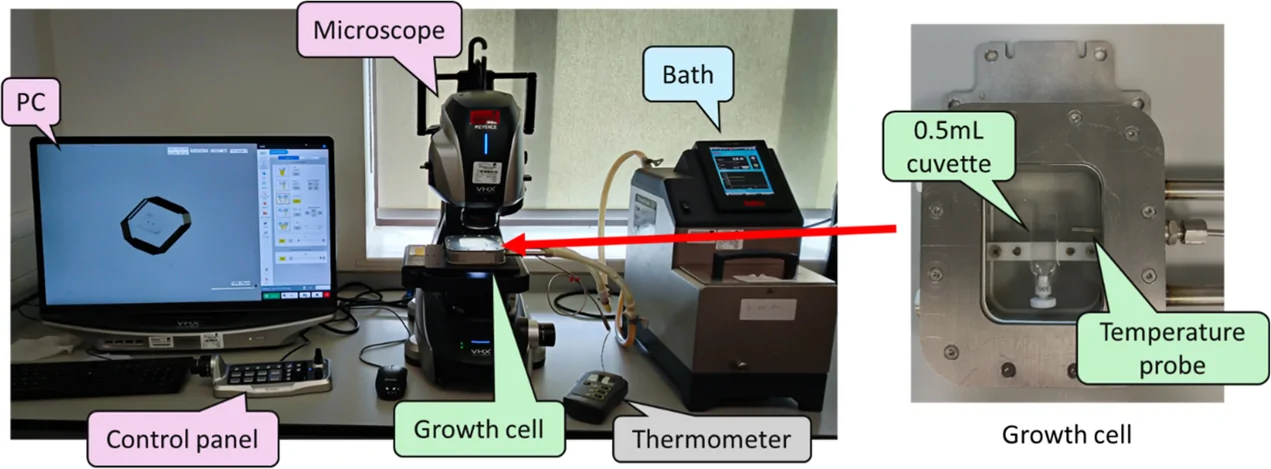
Experimental setup: the growth cell^38^ with a real-time imaging system for α-LGA crystal faceted growth rate measurements. A single crystal seed was placed in the cuvette, which is filled with saturated solution, and immersed in a water bath controlling the temperature of the cuvette. The temperature probe placed inside the growth cell provided an accurate, calibrated measurement of the solution temperature within the cuvette. The KEYENCE microscope^60^ captures images of the crystal inside the cuvette during its growth, with the camera pointing downward orthogonal to the bottom plane of the cuvette on which the crystal rests.

### 2.3 Experimental Methods

#### 2.3.1 Preparation of α-LGA Seed Crystals

Although the α form is the metastable form of LGA, well-defined crystals can be easily prepared by crystallization at lower temperatures.^41^ Such crystals are transparent and typically display well-defined prismatic morphologies.^41^ The solubility of the α form in water has been previously measured by Khellaf et al.,^61^ and this was used in this study to calculate the saturation and supersaturation temperatures. An aqueous solution with 10 g LGA in 1L of water (corresponding saturated temperature around 24 °C) was prepared and heated to around 40 °C until complete solute dissolution was achieved and was then left in an open crystallizing dish at room temperature (around 20 °C) to facilitate slow evaporation crystallization. Crystals typically started to appear within about 12 h before being carefully separated and dried. In order to minimize the potential for size-dependent growth, crystals of a similar size range (ca 500–1000 μm length) were selected as seeds for further growth experiments.

#### 2.3.2 In Situ Growth Rate Measurement

For the in situ crystal growth experiments, LGA aqueous solutions were prepared with 16g LGA in 1L of water (corresponding to a saturated temperature of around 35 °C). The solution was heated to around 50 °C to ensure complete dissolution of crystals and was then transferred into a cuvette, to which a single α-LGA seed was then added. The cuvette was then tightly sealed and cross-checked to ensure no solution leakage or the existence of any air bubbles, which could either cause solvent evaporation or act as secondary nucleation sites. The cuvette was then attached to the fixing clip in the growth cell. Overall, the above steps were carried out rapidly to minimize the time for the hot solution being exposed at the lower ambient temperatures, hence avoiding the potential for spontaneous and secondary nucleation within the cuvette. The growth cell with a pre-set temperature of 35 °C was held between 35–40 °C for a short period of time to allow a limited degree of dissolution of the surface of the seed in order to remove any surface imperfections restricting the potential for secondary nucleation.

The solution was supersaturated by cooling at a rate of 1 °C min^–1^ and held at a fixed temperature of 20 °C. The solution equilibration temperature within the sample cuvette was typically reached in around 15–20 min, allowing the seed crystal to stabilize its facet morphology and exhibit clear and well-defined edges before initiation of the growth process. The equilibration temperature of 20 °C created an initial relative supersaturation σ of 0.78, as defined by

1 $$\sigma =\frac{c}{c_{e}}-1$$

where *c* is the concentration of solution and *c*_*e*_ is the equilibrium saturated concentration of α-LGA at 20 °C, based on the previously published solubility determination.^61^ Temperature within the growth cell was checked regularly during the experiment to ensure that no temperature fluctuations occurred. It should be noted that the experiments were carried out in a stagnant solution environment, where solute mass transfer could occur through free convection without any impact of external driving forces such as agitation.

Crystal image sequences were obtained from the microscopy system, which recorded the α-LGA crystal growth process. The microscopic lens used was VHX-E20 with a magnification of 40× and a nominal focus distance of 30 mm, providing images with a resolution of 2880 × 2160, a field of view of 7.62 mm × 5.70 mm (9.53 mm in diagonal), and a depth of field of 1 mm. Each image sequence contained images of the same crystal, highlighting its temporal development during the growth process. These images were captured at a constant time interval of 5 min, providing a sequence of crystal images, from which the progression of the crystal growth could be observed and measured as a function of time. The growth experiments were typically carried out over a period of ca 15 h, with 180 images being captured for each run. Captured image sequences are shown in Section 4.1. The microscope did not require refocus during image acquisition, as the relatively slow growth on crystal height dimension was not sufficient for the crystal to bypass the depth of field (1 mm) of the microscope.

At the end of the growth process, the cuvette was removed from the growth cell and carefully checked to ensure the absence of other crystals (secondary nucleation). Although unobserved nucleation could, in principle, have occurred during the experiment, such nuclei formed would be too small in size to significantly affect the solution environment or the growth of the seed crystal, and this effect was therefore neglected. The crystal growth experiments were repeated 3 times under the same conditions using similar sized crystal seeds (ca 500–1000 μm in length) to ensure cross-check for their repeatability.

### 2.4 Morphological and Surface Chemistry Characterization

#### 2.4.1 Morphological Characterization

The morphology of α-LGA is well-known and has been well-characterized in the literature.^40,41^ The morphological model of α-LGA used in this study was determined from a combination of both experimental observations and the predicted crystal morphology by Turner et al.,^41^ with the resulting morphology being visualized with SHAPE software.^62^

As the development and application of techniques to determine the size and shape evolution and temporal development of the crystal morphology in 3D, together with the calculation of the associated solution supersaturation, forms a significant component of the outcomes of the research presented in this paper, this is described in detail in Section 3.

#### 2.4.2 Analysis of the Surface Chemistry of Crystal Habit Surfaces

Molecular modelling of the α-LGA crystal structure was carried out using the CCDC’s^58^ Mercury software based upon the structure of α-LGA (ref code LGLUAC02 ^59^) provided in CCDC’s database. Surfaces were analyzed using the VisualHabit morphological tools provided in Mercury with the Dreiding II force field.^63^ The surface chemistry for all the expected morphological forms was also characterized in terms of the density of LGA functional groups exposed at the habit surfaces together with their propensities for intermolecular binding based on the availability of the hydrogen bond (H-bond) donors and acceptors.

#### 2.4.3 Determination of the Crystal Growth Kinetics

The measured face-specific growth rates as a function of the relative supersaturation of the solution were fitted to standard crystal interfacial kinetics models, with the overall mechanisms encompassing consideration of both MT and SI effects based on the following mechanistic model^14^

2 $$G\left(\frac{m}{s}\right)=\frac{1}{\frac{1}{k_{MT}^{\prime }}+\frac{1}{k_{SI}}}\left(\sigma \right)$$

where *G* is the facet growth rate, σ is the relative supersaturation, $\frac{1}{k_{SI}}$ is the resistance of surface integration, and $\frac{1}{k_{MT}^{\prime }}$ is the resistance of mass transfer, satisfying

3 $$k_{MT}^{\prime }\left(\frac{m}{s}\right)=\frac{k_{MT}c_{e}{MW}_{s}}{\rho_{s}}$$

where *k*_MT_ is the coefficient of mass transfer within the bulk of the solution, ρ_s_ is solute density, MW_s_ is solute molecular weight, and *c*_*e*_ is the equilibrium concentration (solubility), and can be calculated from the relative supersaturation σ during growth

4 $$c_{e}=\frac{c_{0}}{1+\sigma }$$

where *c*_0_ is the initial concentration of the solution. In this study, MW_s_ = 147.13g·mol^–1^, ρ_s_ = 1.54g·cm^–3^,^64^ and *c*_0_ = 108.75mol·m^–3^.

In eq 2, the surface integration resistance factor *k*_SI_ can be defined based on the three standard growth mechanism models.

For the power law model^8,9^

5 $$k_{SI}=k_{G}{\left(\sigma \right)}^{r-1}$$

For the Birth and Spread (B&S) model^7^

6 $$k_{SI}=k_{G}{\left(\sigma \right)}^{-1/6}\exp\left(\frac{A_{1}}{\sigma }\right)$$

For the Burton–Cabrera–Frank (BCF) model^6^

7 $$k_{SI}=k_{G}\left(\sigma \right)\tan h\left(\frac{A_{2}}{\sigma }\right)$$

where $k_{G}\left(\frac{m}{s}\right)$ is the growth rate constant, *r* is the growth exponent, and *A*_1_ and *A*_2_ are thermodynamic parameters.

Comparison between the fitted parameters from eq 2 was used to assess whether the overall growth rate on a given crystal habit face was determined (limited) by MT or by SI. Extrapolation of fitting curves from the experimental growth rates also revealed the face-specific critical supersaturation for growth σ_crit_,^14^ below which the growth rate of the face would be expected to be zero.

The data fitting to the models was carried out using Origin Pro^65^ software using the Levenberg–Marquardt algorithm^66,67^ with the quality of the fitting results of the data being assessed through the coefficient of determination *R* ^2^, the adjusted *R* ^2^ (Adj.*R* ^2^), the residual plots, residual sum of squares (RSS), and parameter confidence intervals.

## 3 Determination of Crystal Size, 3D Shape, and Volume

The geometric shape and size of a faceted crystal grown under stable conditions can be represented as a convex polyhedron, i.e., a three-dimensional convex shape displaying flat polygonal faces defined by their Miller indices (hkl). This external morphology can be extracted into a crystal mesh composed of the edges between pairs of crystal habit faces and the corner vertices, where at least three of the faces intersect. Theoretically, by applying this concept, a growing crystal can be represented by varying the shape and size of this mesh. Subsequently, geometric analysis of the fitted mesh can also yield its main features, i.e., face-origin distances, the areas of individual faces, and the overall volume of the crystal, together with changes to these parameters during the crystal growth process.

### 3.1 Crystal Shape Reconstruction from Transmission Images

The overall crystal morphology as represented by a mesh based on the known crystallographic data for α-LGA can be parameterized with a fixed origin point (the nucleation center) and a list of orthogonal distances from the origin to all individual faces. Although the choice of origin can be arbitrary, the origin point must be selected to lie within the expected nucleus of the crystal. If the face-origin distances for all crystal faces in 3D can be measured from an image, then it is possible to reconstruct the full crystal 3D morphology together with its face-specific temporal evolution during the growth process. Through this, the growth of each individual crystal habit face with time can be determined and characterized to yield the growth rate as a function of both the growth environment and kinetic conditions.

In this work, a newly developed software reconstruction tool CrystalSizer3D^57^ has been used to reconstruct the 3D morphology of a growing crystal from a sequence of 2D images taken by a single-view transmission optical microscopy system. The full reconstruction in 3D requires the use of images taken under transmission illumination in order to visualize the structural properties of the bottom-facing faces with respect to the observational direction, as they are necessarily observed through the projected images of the top-facing faces of the crystal. The accuracy of the reconstructed mesh in CrystalSizer3D was evaluated through the examination and cross-calibration of a representative dry α-LGA crystal, whose dimensions were directly measured in 3D by laser confocal microscopy and compared with those predicted using the digital mesh fitted by CrystalSizer3D.^57^ The average error between the CrystalSizer3D reconstructed mesh and laser confocal measurements on each crystal habit face is 11.9%, which is fairly acceptable considering the noisy confocal scanning results on the transparent crystal.^57^

CrystalSizer3D enables the direct estimation of the crystal mesh parameter values from a single 2D image in transmission mode. This is achieved through digital construction of a crystal mesh from a known set of the Miller indices (hkl) that define the external crystal morphology, and projection of the mesh after allowing for refraction effects onto a real crystal image. In the development of CrystalSizer3D,^57^ the refractive index for α-LGA crystal was estimated to be 1.70 through parameter modelling with a solution refractive index of 1.33, which was found to be an optimal value in terms of providing an acceptable refraction when compared to real α-LGA images. At this stage in the development of the analysis software, the optical anisotropic effect due to birefringence effect has been neglected as all tilted habit faces in the digital mesh exhibited perfect refracted edges when projected using a value for the refractive index of 1.70. Through this crystal fitting image analysis approach, the user can fit the digital crystal mesh to the real in situ image by iteratively adjusting mesh parameter values, until the inter-planar facet edges and vertices in the image all align well in a visualization of the projected digital mesh.

The user interface for CrystalSizer3D is shown in Figure 2. From the panel on the left, the user can make adjustments to the parameter values defining the position of the origin point and all face-origin distances for the mesh. The image analysis tool automatically updates the digital mesh and its projection to reflect the changed parameter values. It is worth noting that this approach not only works with α-LGA but is also applicable to other crystal systems. Provided that the crystal unit cell data and refractive index of the material, together with the Miller indices of the expected crystal faces that can be identified, this characterization method can be used to build the mesh, calculate the associated refractions, and perform similar distance measurements for crystals of other compounds.

**Figure 2:**
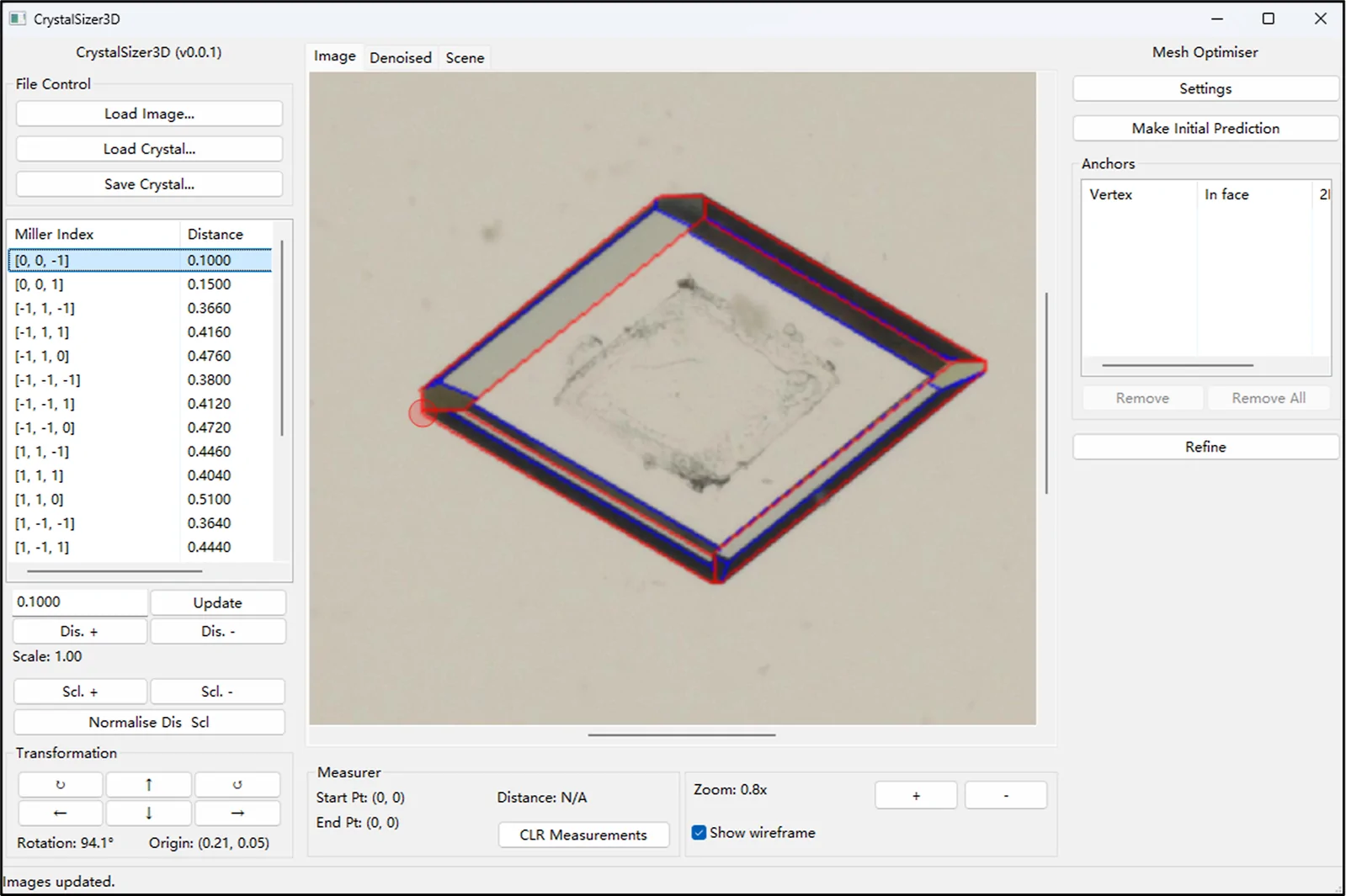
A screenshot showing a typical view of the user interface for the manual fitting mode of CrystalSizer3D,^57^ loaded with a real crystal image on the background and a precisely fitted digital crystal mesh defined in red and blue edges (projected). Reproduced from ref 57 available under a CC BY license. Copyright 2026 Chen Jiang.

While, in principle, this method could be used to process an entire temporal growth sequence, such a process has been found to be too labor-intensive in practice to be used on more than a few images (typically around 20 min to fit a single image). Thus, in this study, 20 images spaced at 50 min intervals were taken from each repeat run for manual fitting. CrystalSizer3D^57^ has two modes of operation: a manual mode requiring interactive alignment of the crystal 3D mesh to a given image, and a fully automatic mode requiring no human intervention. Although the manual mode is more time-consuming, it is also slightly more accurate at this stage of its development within the current version of CrystalSizer3D.

### 3.2 Face-Specific Growth Rates

The growth rate of a habit face is given by the rate of face-to-origin (nucleation center) distance increase, which is independent of the choice of origin (as shown in Figure 3a). The 3D position of the origin does not affect the measurement of facet growth propagation and its associated growth rate, provided its position is fixed during the growth process, as shown in Figure 3a. Also, crystal faces that grow faster relative to their adjacent faces may reduce in surface area or disappear altogether. This process is known as ‘faceting out’ and is illustrated schematically in face *A* in Figure 3b and faces *A* and *B* in Figure 3c. The disappearing faces can be considered to be capped at the facet edge, and hence the perpendicular distance from the origin can still be measured. However, since the face already disappeared, the distance should not be used for growth rate calculation for the face. Reflecting this, crystals of α-LGA were sometimes found to exhibit fewer than the maximum number of 20 faces. Faces that have disappeared may at times also appear again (‘facet in’) if their adjacent faces were growing relatively fast at later stages of the growth process, e.g., face *A* in Figure 3c, in which case these faces could remain in the final morphology, e.g., face *A′* in Figure 3c.

**Figure 3:**
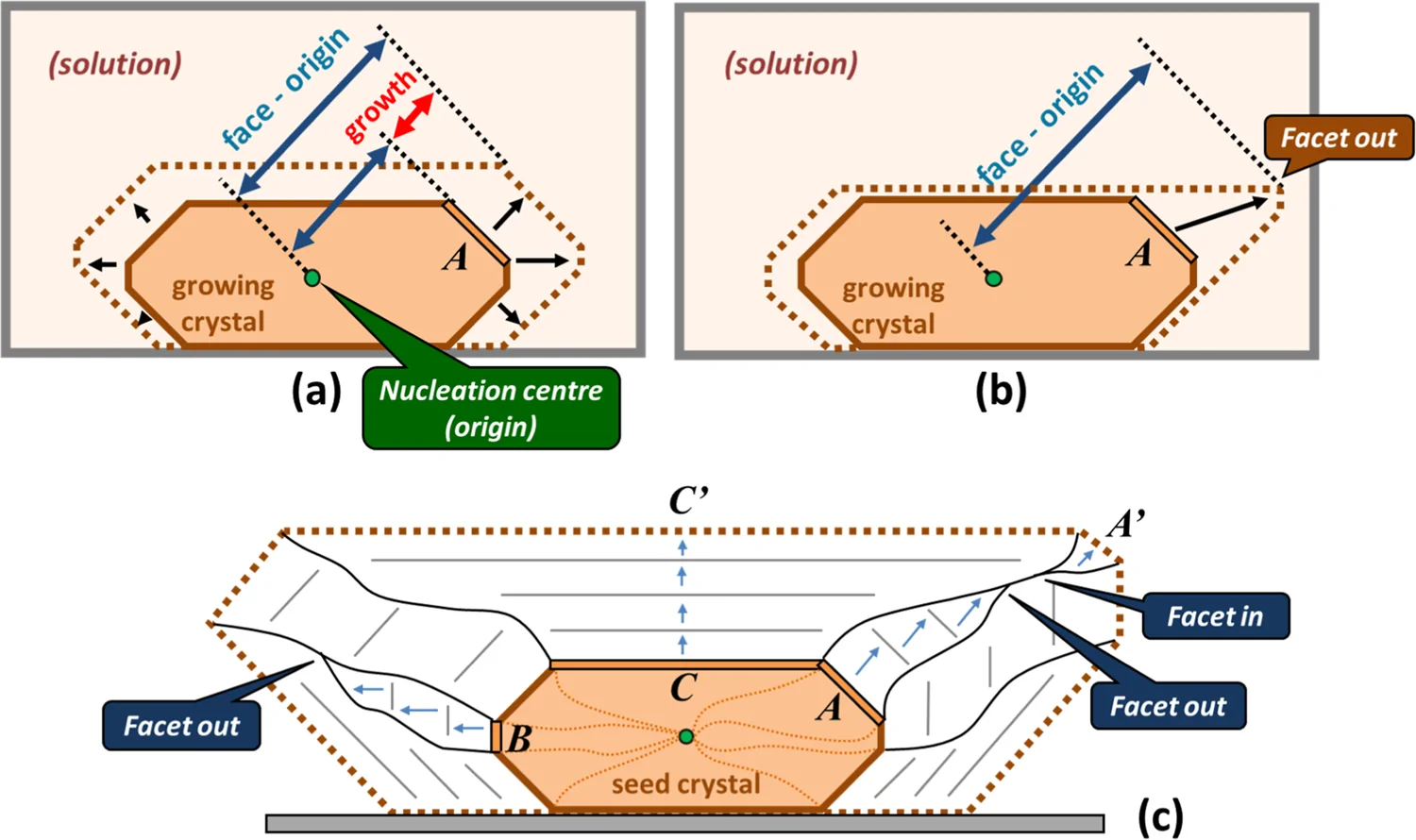
Schematic illustration of the morphological development with time: (a) defining faceted growth in a growing crystal based on an ‘origin’ point; (b) in the case that a face (labeled *A*) is growing too fast and ‘disappears’ from the crystal, face-origin distance can still be measured; (c) the fast-growing surfaces (labeled *A* and *B*) will disappear from the crystal, while slow-growing surfaces such as *C* will have larger area and be morphological dominant in the final morphology (labeled *C′*). The disappeared face *A* may appear again and would stay in the final morphology (labeled *A′*).

Face-specific growth rates at a specific time point were calculated as the slope of the least-squares linear fit on face-origin distances over a sliding window of 3 consecutive fitted frames. The choice of a three-frame window is sufficient to provide a representative growth rate within the defined time-frame interval, while minimizing the influence of the continuously decreasing supersaturation. This also limits the degree of smoothing that would be provided by a wider time window, hence retaining sensitivity to instantaneous changes in growth rates. In practice, it was found that even for the same crystal face, the growth rate may also vary temporally during the growth. This can reflect a number of factors, most notably the decrease in the solution supersaturation as the crystal grows.

### 3.3 Supersaturation Determination during the Growth Process

For the assessment of the growth rate as a function of supersaturation,^38^ the solution supersaturation at any specific time point during the growth process can be determined from the overall volume of the crystal obtained from the fitted 3D crystal mesh, which also provided the areas of individual faces. The surface areas enable assessment as to whether a face has ‘disappeared’ or ‘re-appeared’ during the growth process, as shown in Figure 3. Combining the original mass of solute and the temporally evolving volume together with the crystal’s density, the time-specific relative supersaturation within the growth cell during the growing process can be calculated, thus

8 $$\sigma_{t}=\frac{m_{s}-\rho_{s}\left(V_{t}-V_{0}\right)}{V_{c}c_{0}}-1$$

where σ_*t*_ is the relative supersaturation at time *t*, *m*_s_ is the initial LGA solute mass when preparing solution, ρ_s_ is solute density, *V*_*t*_ and *V*_0_ are the volume of the growing crystal at time *t* and of the seed at the beginning of growth (in the first frame), *V*_*c*_ is the volume of the cuvette, and *c*_0_ is the initial solution concentration at beginning of growth. In this study, *m*_s_ = 8 mg and *V*_*c*_ = 0.5 mL. For α-LGA, ρ_s_ = 1.54 g·cm^–3^.

The volume of the crystal can also be applied to estimate the volume equivalent diameter *d*_*v*_ for the growing crystal in each frame, a parameter which is industrially useful as this parameter is commonly measured with commercial sizing instruments and used routinely in industrial crystallization process monitoring, control, and validation

9 $$d_{v}={\left(\frac{6V_{t}}{\pi }\right)}^{1/3}$$

where *V*_*t*_ is the volume of the growing crystal at time *t*. In this study, for ease of comparison with face-specific growth rates, the volume equivalent radius *r*_*v*_ was applied in place of *d*_*v*_

10 $$r_{v}=\frac{1}{2}d_{v}$$

the growth rate of *r*_*v*_ is also calculated for reference.

## 4 Results and Discussion

### 4.1 Evolution of Crystal during Growth

Figure 4 shows some representative examples from the in situ crystal imaging system, showing the development of the crystals during their growth process.

**Figure 4:**
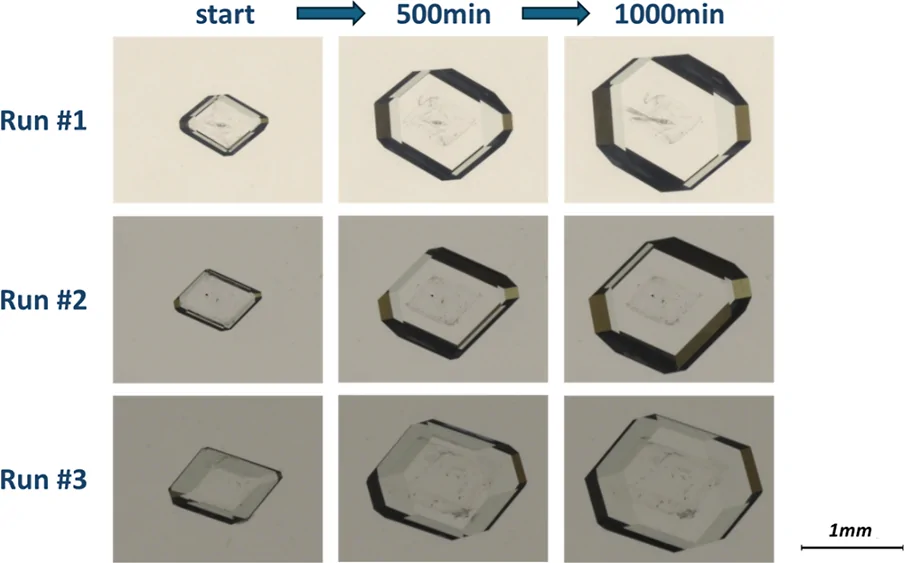
Images taken by the imaging system from different image sequences.

A qualitative examination of the images for each of these sequences reveals that the crystals were growing faster during the earlier stages of the growth process (0–500 min) when compared to the later stages (500–1000 min). This is consistent with the decrease in supersaturation inside the cuvette during crystal growth.

### 4.2 Morphological Characterization

A schematic of the crystal morphology of the α form of LGA is shown in Figure 5, revealing that the morphology can be characterized by 6 crystallographically unique morphological forms. These, together with their respective multiplicities, encompassed a total of up to 20 faces, as detailed in Table 1. Note that due to the non-centrosymmetric crystal structure of α-LGA, the {111} and {-1-1-1} are polar forms and are not crystallographically equivalent.

**Figure 5:**
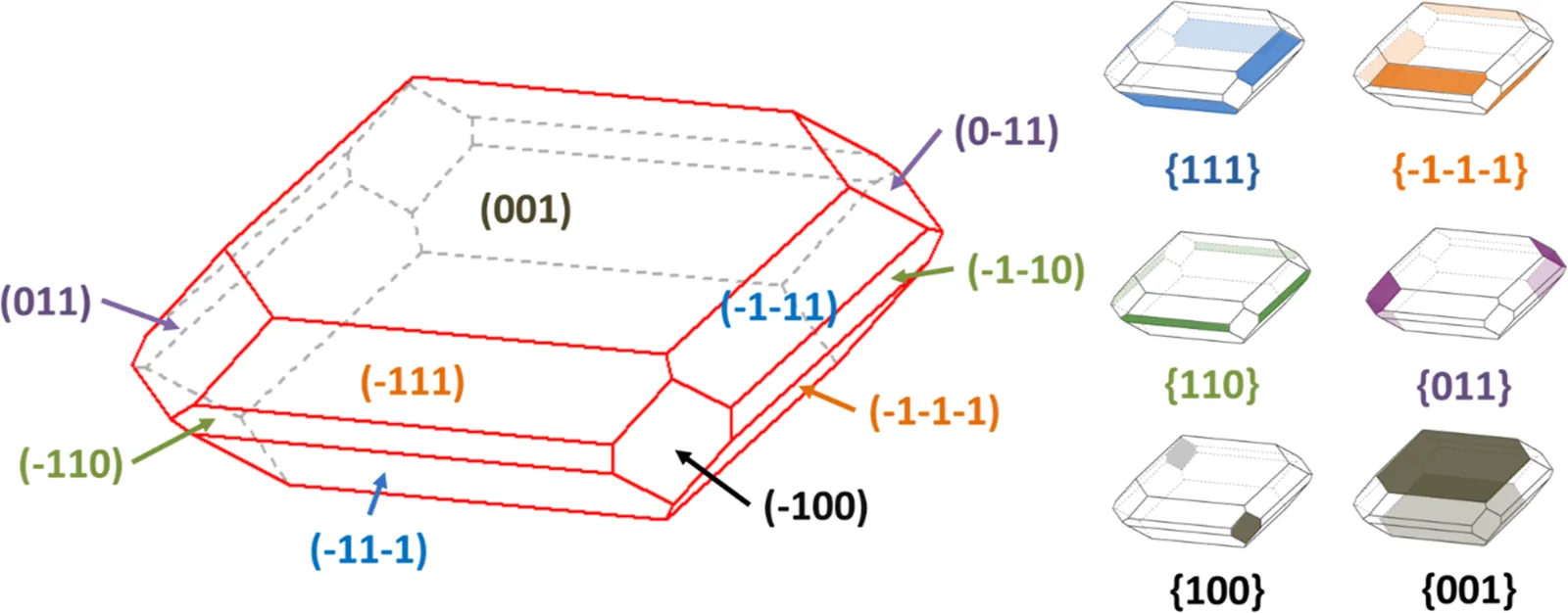
Schematic representation of the crystal morphological mesh for α-LGA revealing the crystal habit surfaces associated with the 6 morphological forms (plotted with SHAPE software^62^).

**Table 1:**
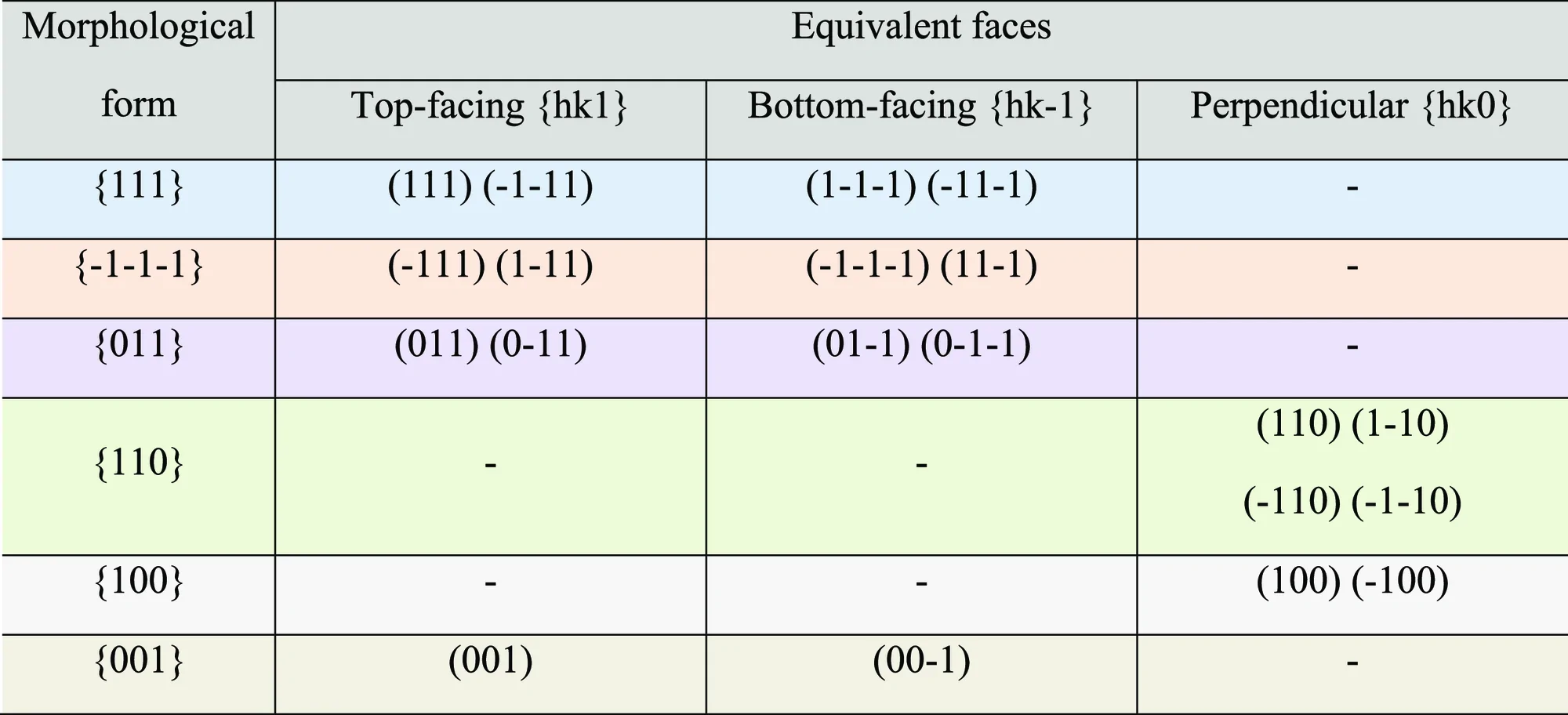
List of Morphological Forms and Crystallographically Related Faces for the α-LGA Crystals, with the Faces Being Sorted by Their Facing Orientation with Respect to the Growth Cell as Shown in Figure 6a

Overall, the α-LGA crystal morphology was found to be in good agreement with that predicted from the morphological analysis presented by Turner et al.^41^ In this, the {001}, {111}, and {-1-1-1} faces tend to dominate in the final crystal morphology, with the smaller surface area {100}, {110}, and {011} faces also being present. The {100} faces were not considered in the morphological predictions of Turner et al.^41^ because of their relatively low *d*-spacing value among all the possible forms in the attachment energy morphology.

### 4.3 Correlation of Digital Crystals with Crystal Images

Experimental observations revealed that the crystal seeds placed in the cuvette tended to lie on their largest habit face {001} on the bottom of the cuvette such that the growth of the lower face (00–1), which is in direct contact with the glass surface of the cuvette, would be negligible. Hence, in this arrangement, all the top-facing faces {hk1} were directly visible for the camera (shown as the red colored lines in Figure 6c) with the faces perpendicular to the imaging plane only being seen as edges in the image. As α-LGA crystals are transparent, the bottom-facing faces {hk-1} can only be seen through the refraction effects on the top faces due to the transmission lighting (shown as the white dashed lines in Figure 6c) provided by the microscope.

**Figure 6:**
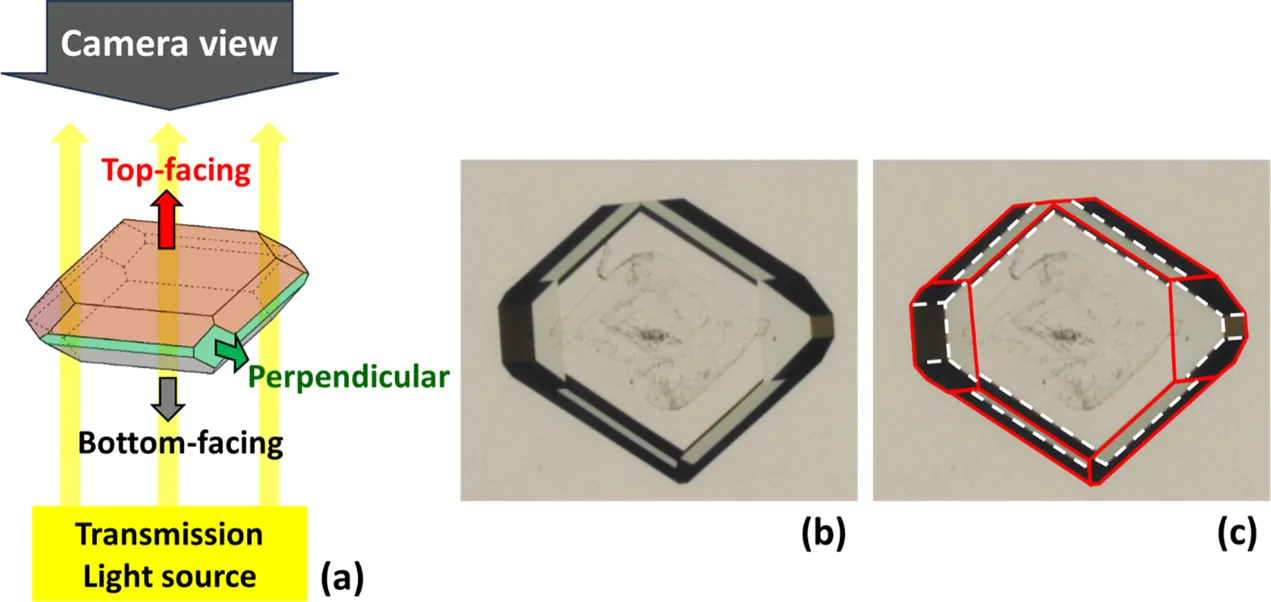
How different faces are imaged by the camera: (a) schematic highlighting top-facing faces (red), perpendicular faces (green), and bottom-facing faces (gray); (b) an original α-LGA crystal image; (c) crystal faces have been identified from the image: red solid edges are from top-facing faces {hk1} and are directly visible, white dashed lines are from bottom-facing faces {hk-1}, refracted by top-facing faces.

Because most faces in α-LGA are neither parallel nor perpendicular to the imaging plane, they are not captured at their true size in the image due to perspective distortion effects of the transmission optical geometry. Hence, it was not feasible to measure the growth of these faces directly from the observed 2D crystal images. However, for the bottom-facing faces {hk-1}, their refracted images of the edges still contain a lot of facet surface information from which their shape and position can be ascertained through modelling, which then enables characterization of the 3D shape of the entire crystal.

Figure 7 shows a digital crystal mesh with predicted refractions created by CrystalSizer3D. The mesh structure undergoes refraction at the top-facing faces, which results in some crystal edges or vertices from the bottom-facing faces being visible more than once, as shown in Figure 7a, which can also be seen in real images such as Figure 7b.

**Figure 7:**
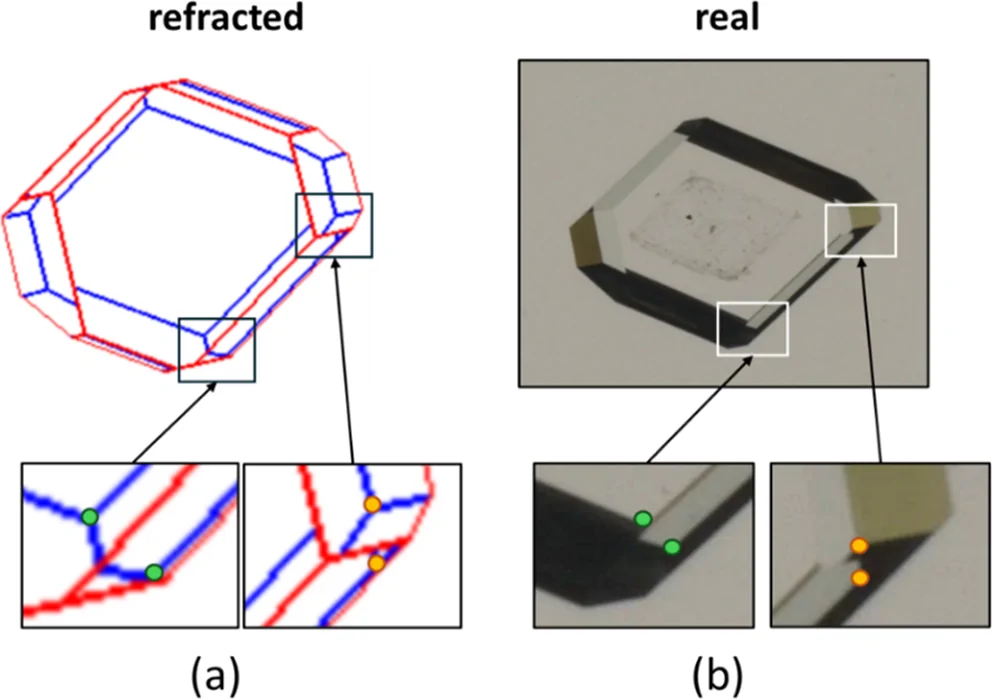
(a) A projected digital crystal mesh with refraction of bottom-facing faces: red solid lines are edges of the directly visible top-facing faces and blue solid lines are refracted edges of bottom-facing faces. The partial images on the insets at the bottom show two of the bottom-facing vertices that can be seen twice. In each case, the pair of green/orange dots corresponds to the same back vertex that has been refracted through two different front faces. Similarly, the images of the bottom-facing edges that intersect at these vertices can also be seen twice; (b) a real transmission crystal image showing the same phenomenon.

### 4.4 Determination of Facet Growth Rates vs Supersaturation and Growth Kinetic Mechanisms

The original data of measured face-origin distances for all the crystal habit faces for the 3 repeat runs are given in Figure S1 (Supporting Information), with all the associated growth rates for all the selected frames being calculated as described in Section 3.2.

#### 4.4.1 Facet Growth Rates vs Supersaturation

Theoretically, the crystallographically equivalent faces belonging to the same crystallographic form would, in general, be expected to exhibit similar growth behavior. However, in practice, all these faces were found to grow at individual rates, even for the crystallographically equivalent faces. In particular, some top-facing faces {hk1} and bottom-facing faces {hk-1}, despite being crystallographically equivalent, were observed to display distinctly different growth behavior. Hence, the growth rates for the case of these ‘crystallographically equivalent but differently oriented’ faces with respect to the growth environment in the growth cell were analyzed separately. For faces not affected by this situation, i.e., the perpendicular faces highlighted in Figure 6, the average growth rate of all equivalent faces from all 3 repeat runs was taken for each specific crystallographic form. Further discussion of this aspect will be given in Section 5 on GRD.

For each frame in the sequence, the average relative supersaturation was calculated across all three repeat runs as described in Section 3.3 (and shown in Figure S2). Therefore, a list of growth rates against the corresponding relative supersaturation for the same frames was obtained for growth kinetics analysis. The calculated faceted growth rates versus relative supersaturation for each of the repeat runs are given in Figure S3. The growth rate measurement results for all the habit faces in an α-LGA crystal under the range of calculated supersaturations are shown in Table 2, Figure 8, and Table S1. As discussed earlier, the growth rate for the (00–1) bottom face was fixed at zero, consistent with its expected minimal growth rate, as defined in Section 4.3, and was thus not used in growth kinetics analysis.

**Table 2:**
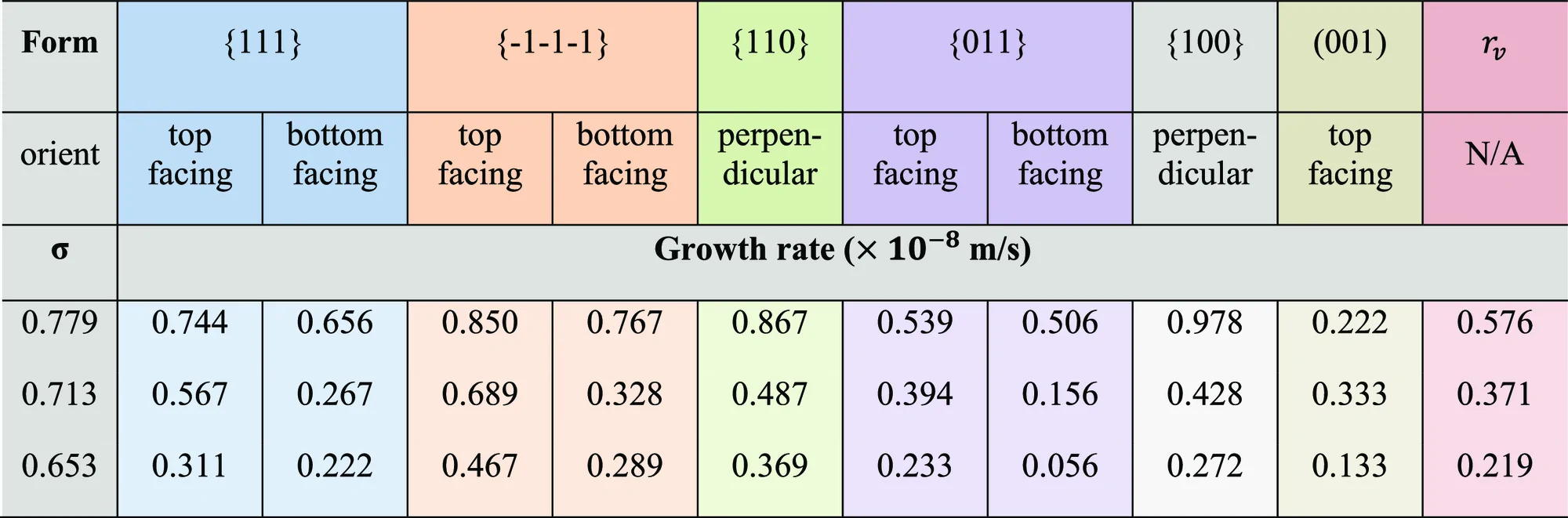
List of Averaged Growth Rates from Measurements for all Crystallographic Forms under Different Relative Supersaturations

**Figure 8:**
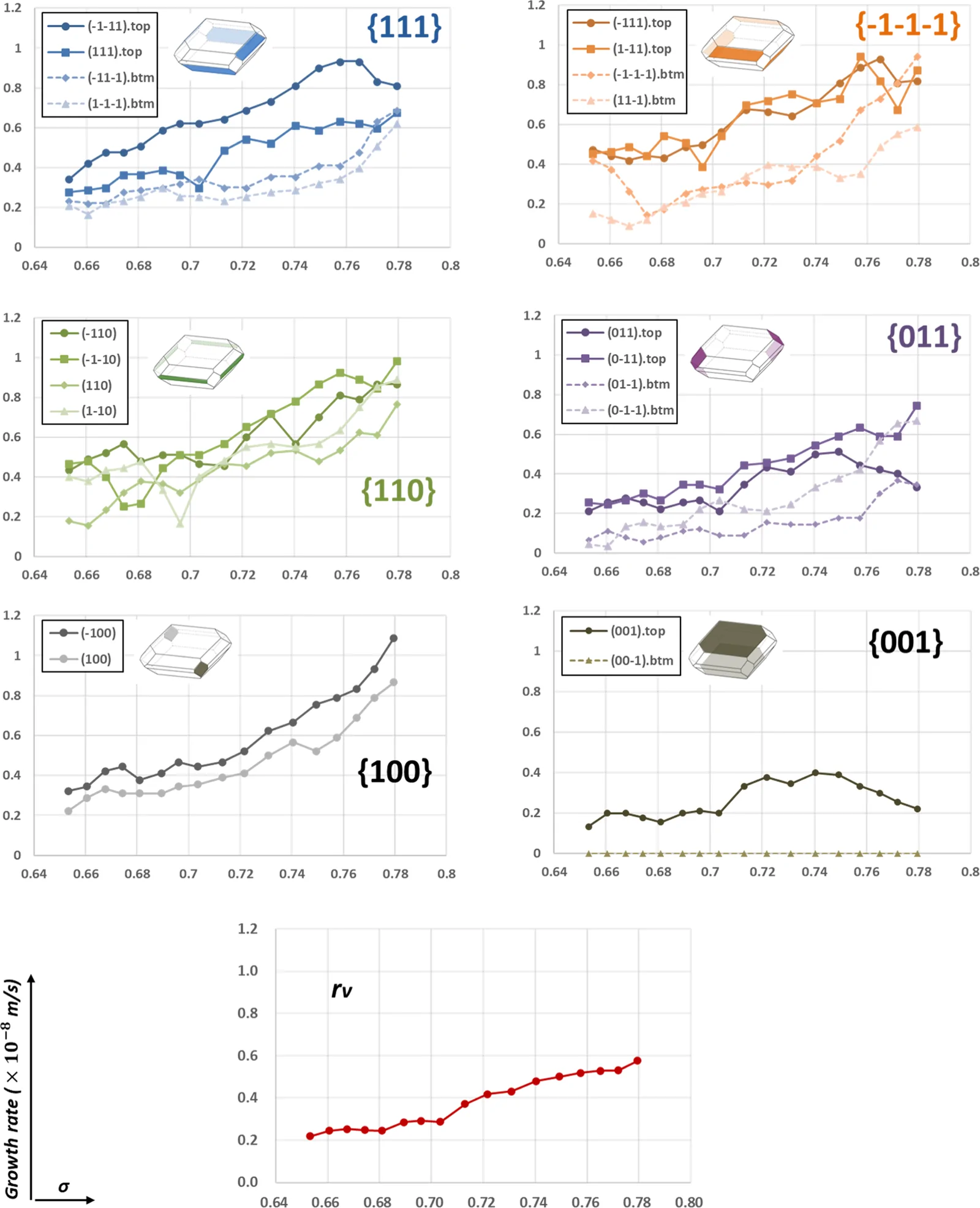
Growth rate measurement results grouped by crystallographic forms. In {111}, {-1-1-1}, {011}, and {001} forms, the legend shows which orientation the faces are ‘top’ for top-facing and ‘btm’ for bottom-facing.

For completeness, analysis of the crystal growth rate on the basis of the volume equivalent radius *r*_*v*_ of the whole particle, on the other hand, provided an assessment of the overall growth for the entire crystal. This analysis is at much lower granularity and arguably hides its fine detail, provided by face-specific measurements. Nonetheless, the data are potentially useful, mindful that volume equivalent radius/diameter crystal size assessments are routinely carried out for quality control in the industrial environment and hence provide a useful comparative metric.

#### 4.4.2 Facet Growth Kinetics Mechanisms

The growth kinetics for each crystallographic form, as obtained by fitting the growth rate as a function of relative supersaturation data for the 3 kinetic models, are given in Table 3, Figure 9, and Table S2.

**Table 3:**
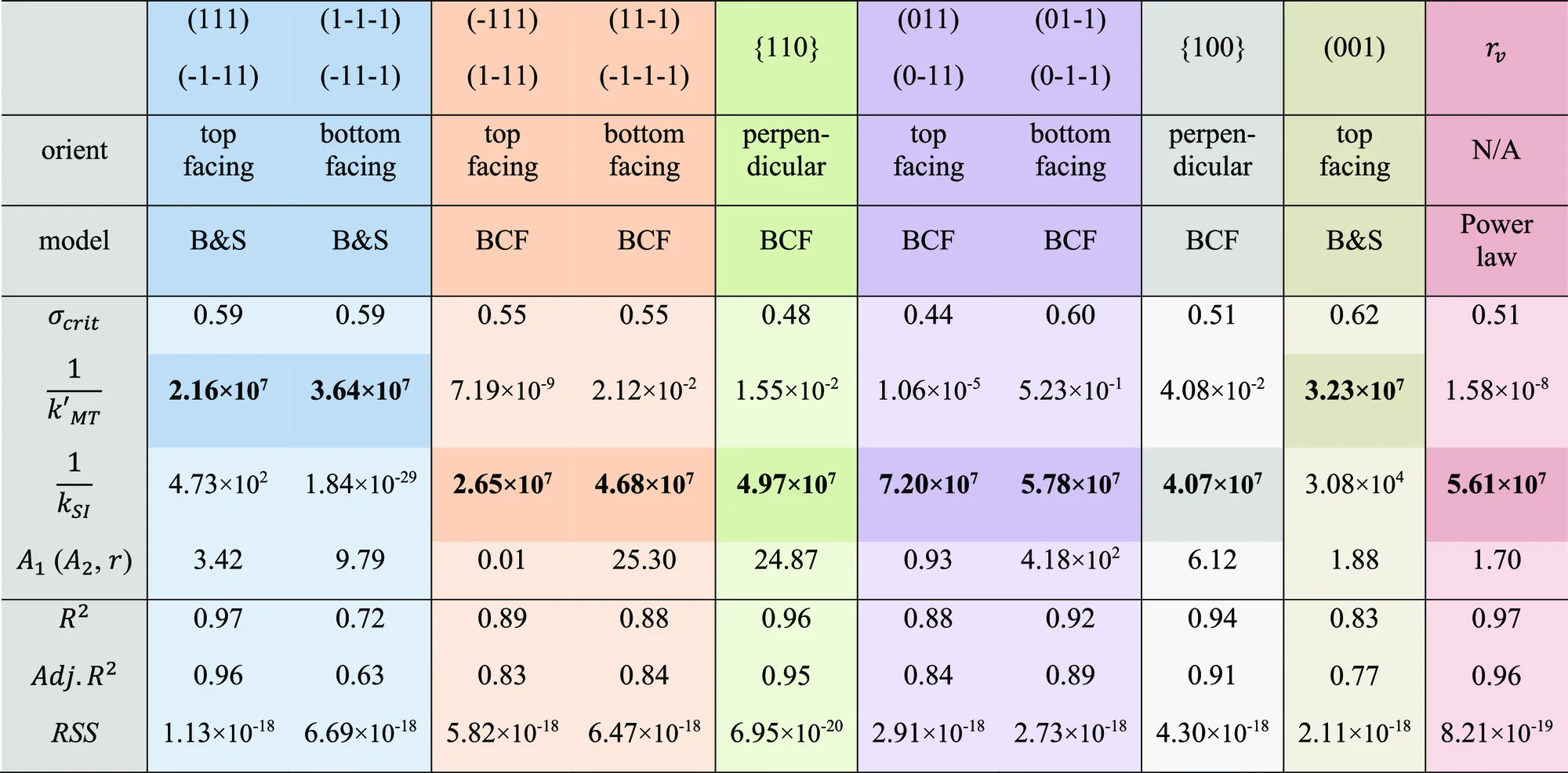
List of Parameters of Faceted Growth Kinetics from the Best Fitting of Experimental Growth Rates with the Kinetic Models^a^

a Resistance of mass transfer $\frac{1}{k_{MT}^{\prime }}$ and surface integration $\frac{1}{k_{SI}}$ was calculated with the average relative supersaturation within the measured range σ̅ = 0.712. Note that bold background colour is indicative of the rate-determination step.

**Figure 9:**
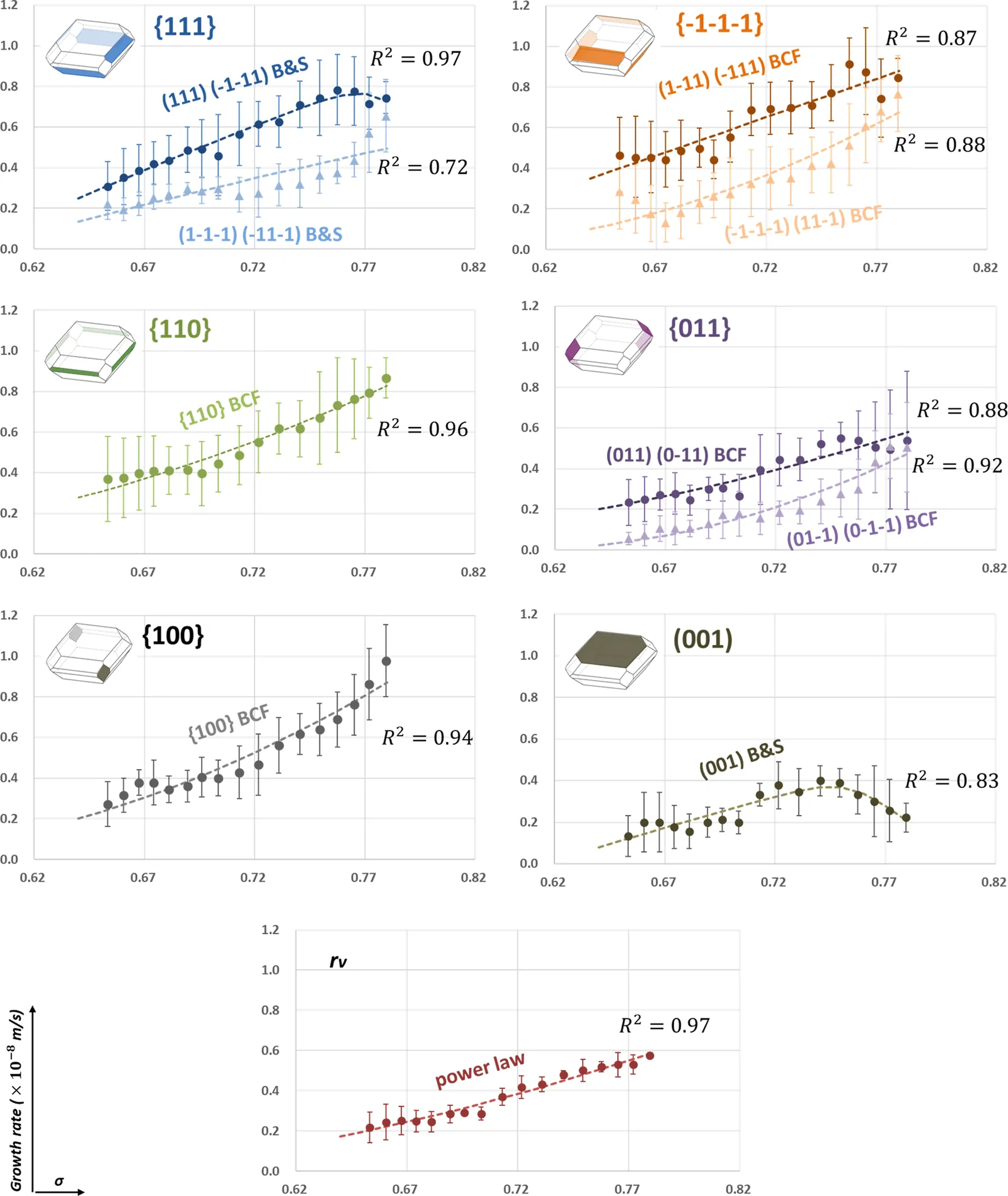
Plot of averaged growth rates on different faces (symbols) along with the curves of their best-fitting kinetic models (dashed lines) and fitting *R* ^2^. Error bars show the standard deviation for each measured point.

The best fit of the 3 kinetic models for each morphological form was selected based on higher *R* ^2^ and values, together with a lower RSS, indicating the closest fitting results to the original growth rate data. It can be seen that most of the forms in the crystal have the best fit to the kinetics based on the BCF model, apart from those for the {111} and (001) faces, which have a better fit with the B&S model (assuming that the crystallographically equivalent faces grow under the same mechanism). The confidence intervals for the system parameters, as provided in Table S2, demonstrate the overall stability of the parameter estimation, while the residual plots provided in Figure S4 confirm the reliability of the fitted model with no significant systematic trends being found in the associated errors.

The simpler and more approximate power law model was not considered for most forms mindful that it neglects the surface integration mechanisms taking place during growth. However, as a comparison, this model was used to model the growth rates based upon the volume equivalent radius *r*_*v*_, consistent with the industrial approach of analyzing growth mechanisms.

Closer examination of the growth rate of the (001) surface reveals significant scattering of data points with little variation with increasing supersaturation (Figure S3), possibly reflecting the small supersaturation range available in this study. The sluggish growth observed on this largest surface in the crystal habit is consistent with a B&S mechanism but mechanistically one that perhaps becomes hindered by crystal-size-dependent growth effects (see, e.g., ref 68). This supports the view that this highly morphologically stable face possesses a large dead zone in which the local supersaturation is insufficient to initiate two-dimensional nucleation at the facet center. Consequently, nucleation would be more likely to occur at the facet edges through an overall growth process that remains quite slow. In this case, the crystal surface area increases in size, and solute MT to the central region of the facet becomes progressively less effective. Such a reduction in solute supply would be expected to limit surface step advancement and could eventually lead to stagnation of growth at the facet center. Such a situation would also be compounded by the absence of screw dislocations capable of providing a continuous source of growth steps on facet surfaces. For the (001) surface, such dislocations would require the presence of a [001] Burgers vector, which has a high energy of formation due to the long lattice spacing normal to the {001} crystal habit plane.^69^ As a result, screw dislocation sources would be unlikely to form or persist, further suppressing growth at the center of this face.

Detailed examination of the resistance of MT ( $\frac{1}{k_{MT}^{\prime }}$ ) and surface integration ( $\frac{1}{k_{SI}}$ ) inherent in eq 2 was indicative of the rate-limiting step in the two-stage growth process, i.e., whether MT or SI displays greater resistance to the growth process and can be taken as the rate limiting step in terms of the growth rate of each face. This is highlighted in bold for each form in Table 3. From this, it can be seen that most forms exhibit the SI limited growth apart from {111} and (001) faces, which were formed to be consistent with MT limited growth.

#### 4.4.3 Comparison with Previously Published Growth Rate Data

In comparison with previous and published studies on α-LGA, the growth rates measured on {111}, {001}, and {011} faces in this study fall within the same order of magnitude when compared with previous work on α-LGA by Kitamura et al.^42,43,55^ (as shown in Table 4). Kitamura et al. measured the 2D growth rates along the growth normal directions of [110], [010], and [001] for a single α-LGA crystal and calculated the growth rates for (111) and (011) faces based upon the known angles between the face and the measurement direction.^42,43^ This approach provided an indirect estimation of the growth rates rather than the more direct measurement approach adopted in this study.

**Table 4:**
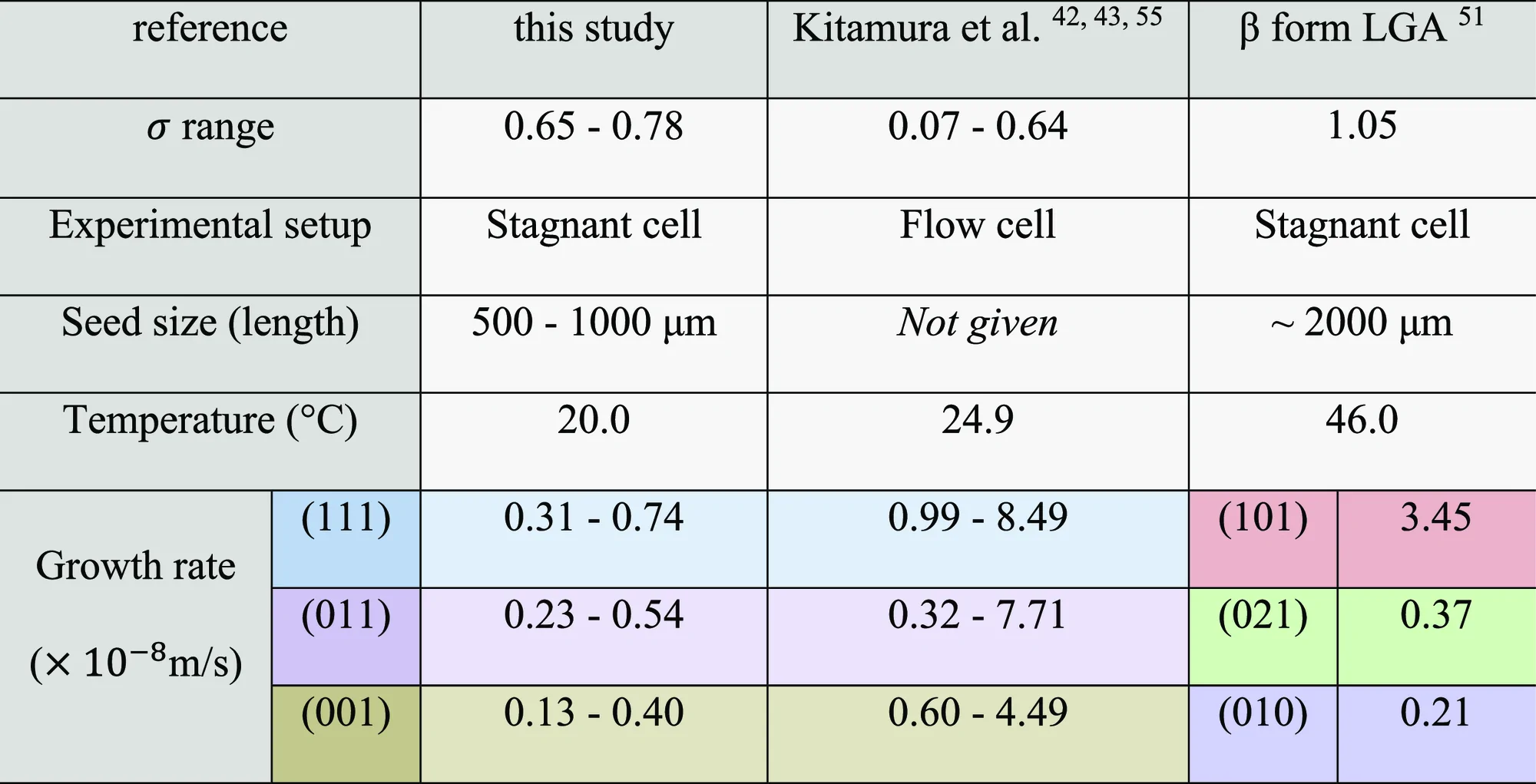
Comparison between Facet Growth Rates for α-LGA Crystals from This Study and Previous Studies

Kitamura et al.’s results showed higher growth rates on all measured faces than observed in this study, reflecting perhaps the higher crystallization temperatures that were studied and also differences in the size of the seeds. It is worth noting that Kitamura et al.’s experiments were also conducted in an experimental setup under flowing solution conditions,^42^ where the seed was secured in the cuvette with solution flowing through, therefore mitigating the effect of supersaturation decrease during growth and enhancing MT.^43^ Despite this, Kitamura et al.’s atomic force microscopy observations still confirmed that the growth mechanism for faces (111) and (001) is due to two-dimensional nucleation, i.e., the B&S model, which is consistent with this study.^55^

The facet specific growth rate for the stable β form of LGA has also been measured in previous studies,^51^ as shown in Table 4. The β-form LGA crystals exhibit a needle-like morphology, with {101} as the capping faces that have extremely high growth rates and {010} as the slowest growing morphological dominant face. It can be seen that the difference on growth rates for different faces in β-LGA crystals is much more significant than that of α-LGA. This results in more obvious morphology change during the growth of β-LGA crystals. The latter eventually leads to the long, thin needle-like morphology for the as-grown β-LGA crystals when compared to the prismatic morphology of α-LGA crystals showing a lesser degree of morphological development during the growth process. However, at σ = 0.78, the growth rates for α-LGA crystals on most faces are higher than those in β-LGA crystals apart from the {101} faces. This is the case even for the β-LGA crystals that were grown at a higher supersaturation of 1.05. Overall, this result is not surprising mindful that such higher growth rates would be consistent with the metastability of the α form.

### 4.5 Growth Rates of the Crystallographically Independent Habit Surfaces in Relation to Their Surface Chemistry

The surface analysis results conducted in this study, together with data regarding solvent and solute binding interactions from previously published work by Turner et al.,^41^ are summarized in Table 5 and Figure 10.

**Table 5:**
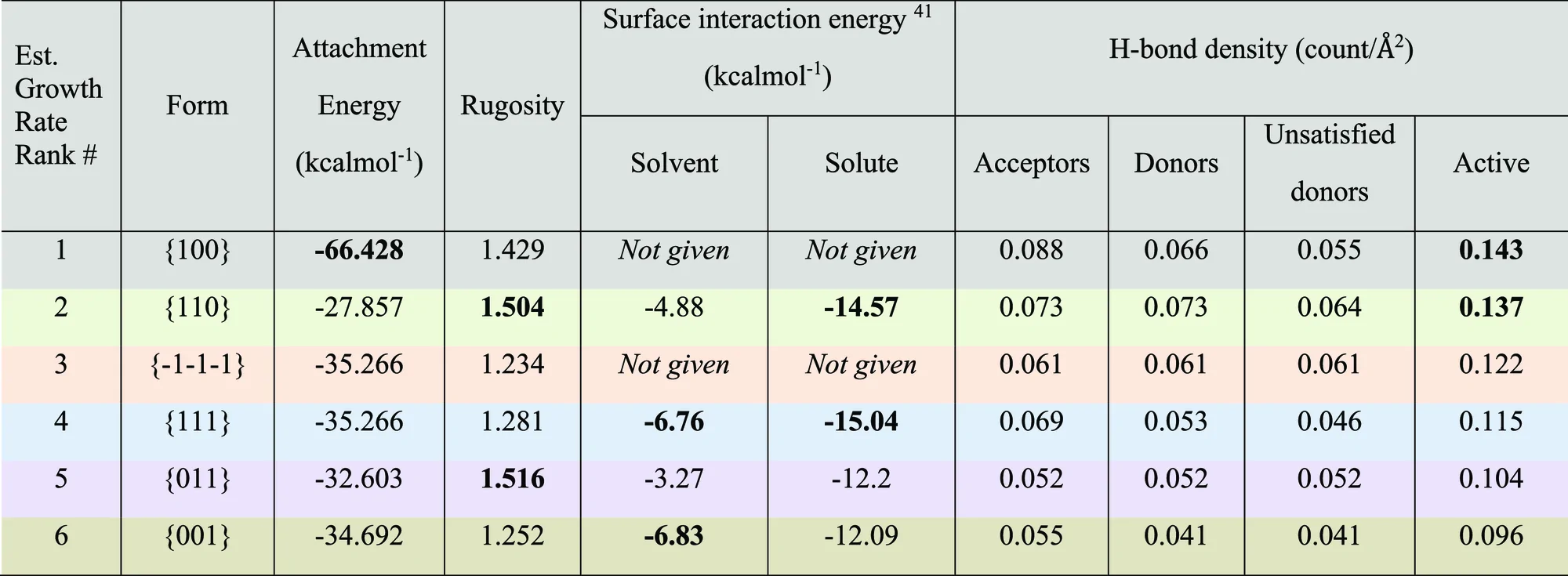
Surface Analysis Results by Different Forms^a^

a Solvent and solute interaction energy data are adapted from the literature.^41^ The ‘active’ H-bond density means the sum of H-bond acceptor density and unsatisfied H-bond donor density. Forms are ranked by their estimated growth rate based on surface chemistry, from fast to slow. Slower-growing forms are more likely to be morphology-dominant at the end of the growth process.

**Figure 10:**
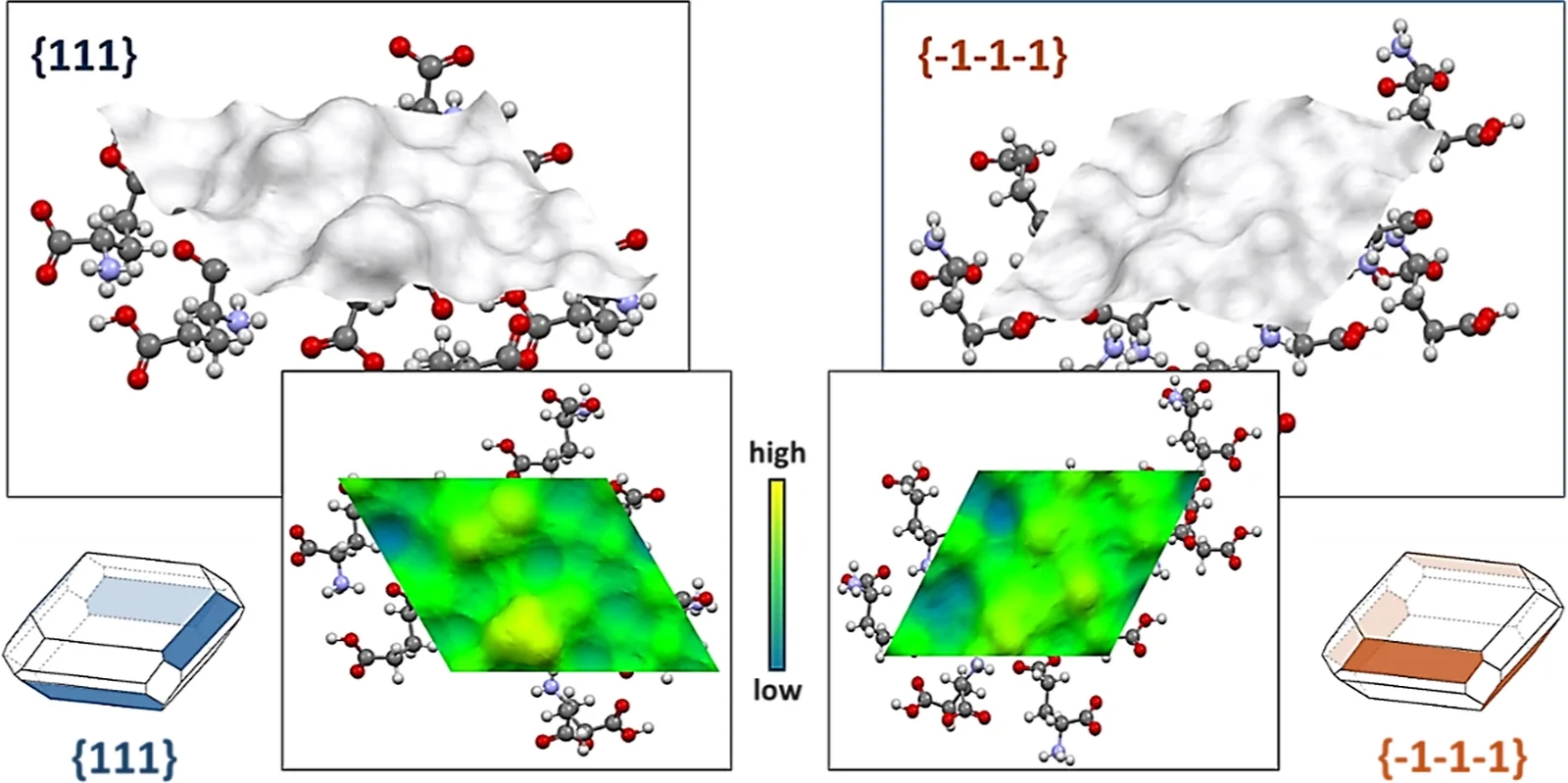
Difference of surface topological structure between non-crystallographically related polar forms {111} and {-1-1-1}. The {111} and {-1-1-1} forms^41^ show similar surface characteristics, with rugosity values in the range between 1.234–1.281 with H-bond densities in the range of 0.115–0.122, indicating they should be extended to exhibit similar growth rates.

It can be seen that the attachment energies for most of the crystallographic forms are quite similar, except for the {100} form, which is significantly higher, consistent with it having the highest growth rate within the crystal. The {110} and {100} forms were found to have the highest density of active H-bonding sites, which would suggest that they might have a higher growth rate when compared to the {011} and {001} forms, which have lower growth rates and a concomitantly lower density of active H-bonding sites.

Estimation of solvent and solute interaction energies previously published by Turner et al.^41^ was only for 4 of the 6 forms considered here. Nonetheless, it can be seen that the {111} and {110} forms have higher solute interaction energies, which suggests that they would have overall higher growth rates. However, despite the {111} and {110} forms having similar solute interaction energies, the {111} form exhibits notably higher solvent interaction energies, which is consistent with the reduced growth rate for {111} faces compared to {110} faces. Similarly, the {011} and {001} forms have overall lower solute interaction energies and growth rates, and {001} would be expected to have lower growth rates than {011} because of its higher strength of solvent interactions.

Forms {111} and {-1-1-1} are polar surfaces and hence represent two different forms reflecting the fact that the LGA molecules are packed in the non-centrosymmetric space group *P*2_1_2_1_2_1_, resulting in the surface chemical structure exposed on {111} and {-1-1-1} surfaces being quite different, as shown in Figure 10. However, the two forms {111} and {-1-1-1} do show similar values in terms of H-bond density, which would suggest that they should exhibit quite similar growth rates. Analysis of the active H-bond density suggests that the {-1-1-1} form may have a slightly higher growth rate than the {111} form. However, they were both found to be less growth-promoting when compared to, e.g., the {110} and {100} surfaces, consistent with them being more likely to be present in the final expected crystal morphology. Further details regarding the surface topological structures for all the forms are given in Table S3.

Overall, the results are in good agreement with the measurements from facet growth rates in Section 4.4. The {001} form is the overall slowest-growing form, enabling it to be the morphologically dominant surface in α-LGA crystals. The {111} and {-1-1-1} forms have similar growth rates, while {-1-1-1} faces grow a little faster than {111} faces, which makes the areas of {-1-1-1} faces usually smaller than {111} faces. This can actually be seen in Figure 4. The {110} and {100} forms have the fastest growth rates among all forms, which makes them more likely to disappear in the final crystal morphology.

## 5 Crystal Growth Rate Dispersion

GRD was observed in different aspects in this study, temporally and in terms of both the different crystallographically equivalent faces and the same face in different experiments.

### 5.1 Observations on Crystallographically Equivalent Faces

Different growth rates were exhibited among the habit faces of the same crystallographic form, as shown in Section 4.4, consistent with variations in the solute MT processes for these equivalent faces due to the nature of crystallization environment.

This effect was especially noticeable in comparison to the top-facing faces {hk1} and bottom-facing faces {hk-1} for the {111}, {-1-1-1}, and {011} forms, which exhibit quite different growth behaviors in Table 2 and Figure 8. This reflects perhaps the design of the growth cell system, where the top faces are probably more exposed to efficient MT in the solution than those on the bottom faces. As shown in Table 3 and Table S2, for all forms with the ‘top- or bottom-facing’ effect ({111}, {-1-1-1}, and {011}), the MT resistance is always higher on bottom-facing faces (hk-1) than top faces (hk1) within the same form, with the differences sometimes being several orders of magnitude.

This indicates that the MT process may have a notable impact on the differences between the growth behavior of the top and bottom faces, with solute diffusion to the bottom faces having a higher resistance when compared to the top faces and thus affecting their respective growth rates. This is a reasonable deduction, mindful of the stagnant solution environment within the cuvette and the small gap space between the bottom-facing crystal habit surfaces and the cuvette base, which would restrict solute diffusion to these areas. The case is the same for the (11–1), (-1-1-1), (01-1), and (0-1-1) faces, even though their growth was not found to be MT rate-limited.

To the best of our knowledge, similar behavior has not been characterized in this level of detail before, as most previous studies usually simply regarded the crystallographically equivalent faces within the same form to have the same growth rates^38,42^ or have neglected the potential impact of the local crystallization environment on the growth process, in this particular case, the top versus bottom-facing effect.^51^ Previous studies by Nicholson et al.^10^ found that the increase of relative supersaturation led to a thicker boundary layer, but this was offset by the increased mass flux of solute within the boundary layer, with the growth rate being eventually limited by the surface integration process due to the undersaturated solute concentration on the crystal surface.

### 5.2 Observations on the Same Habit Face in Different Crystals

Examination of the temporal growth development data reveals that GRD was also observed for the same habit face for the 3 different runs, as shown in Figures 11 and S5. Dispersion in α-LGA crystal growth data due to the effect of using different seed crystals has also been previously observed by Kitamura et al.^42^ This perhaps suggests that slight differences in the shape and size of the seed crystals might lead to deviations in growth rate measurements. It can also be seen that the growth rate from the same run does not always decrease with the growth process, such as run #2 in Figure 11. This may reflect the complexities associated with the cooperative mechanism of the growth of neighboring faces and the adjacent habit faces, e.g., the different faces competing for solute binding.

**Figure 11:**
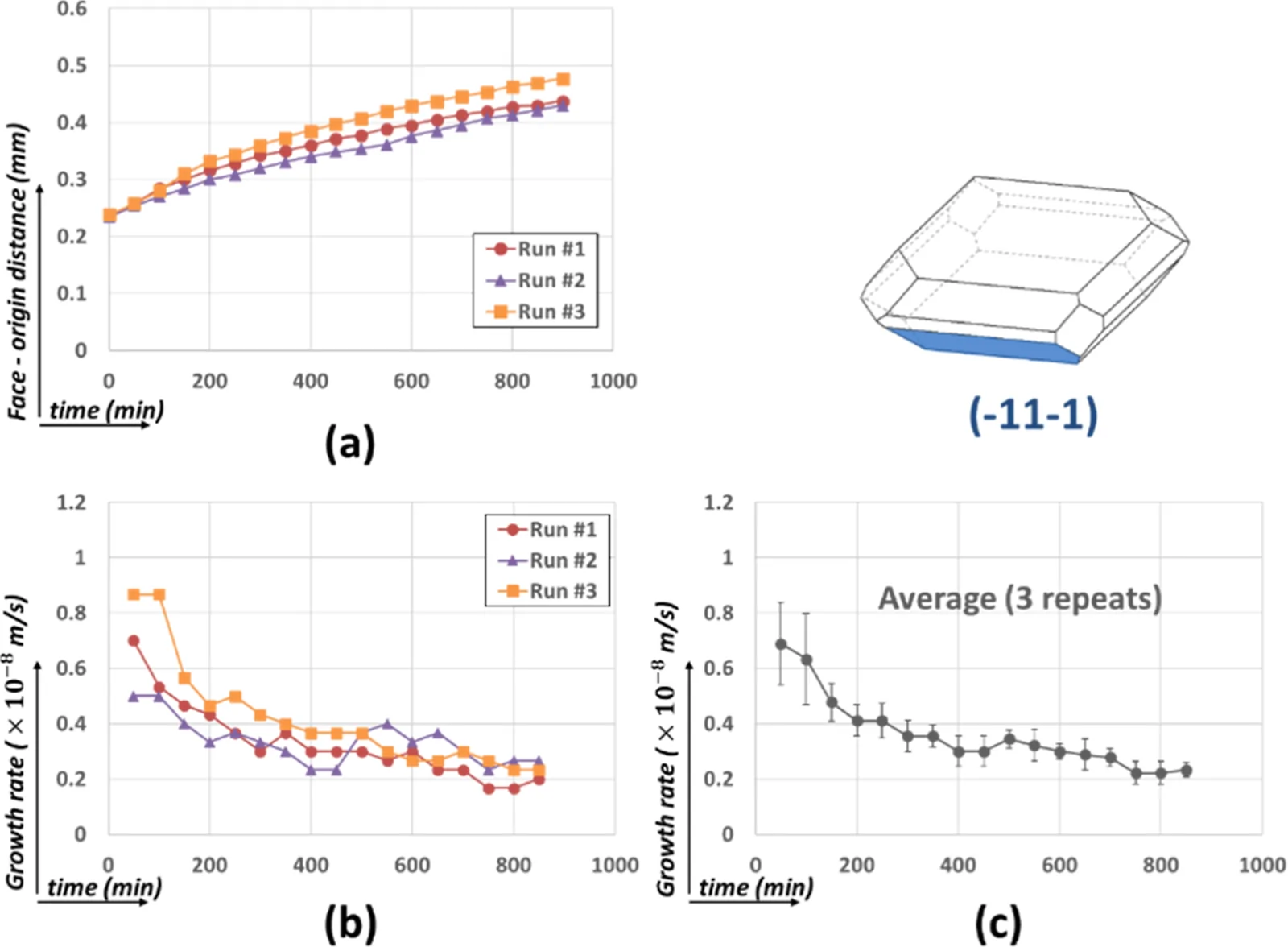
Growth rate dispersion on habit face (-11-1) in 3 repeat runs: (a) the face-origin distances vs time for 3 repeat runs; (b) the growth rate vs time for 3 repeat runs; and (c) the averaged growth rate among 3 repeat runs; error bars represent standard deviation.

The exact reason for this type of GRD remains unclear. Previous studies have proposed that growth dislocations may play an important role in influencing crystal growth behavior. Sherwood et al.,^32,33^ for example, found that the growth rates of individual crystal surfaces were strongly affected by the presence of both edge and screw dislocations, with higher dislocation densities generally leading to higher growth rates. This might affect habit faces that grow under the BCF mechanism, which requires the presence of screw dislocation step sources on their surfaces. Other related studies have also suggested that the presence of lattice strain within the crystals can also have a significant effect, with the more highly strained crystals tending to grow slower than unstrained ones.^34^ Hence, GRD may be regarded as a complex combination of both lattice strain and dislocations, with lattice strain being the main cause for the anomalous existence of size-dependent growth behavior within the smaller crystals of the crystal size distribution in industrial crystallization processes.^35^ This could potentially influence those habit faces that grow under the B&S mechanism, i.e., those that mainly rely on the generation and development of 2D nucleation.

Therefore, tiny differences in crystal perfection on the same habit face of different crystals could, in principle, lead to an unpredictable dispersion of the crystal growth rates. However, the exact fundamental mechanism is unclear currently, and further detailed experimental studies are clearly needed on the impact of lattice defects on GRD.

## 6 Conclusions and Future Perspectives

The growth rates for all faces of α-LGA were measured using sequences of images captured from a temperature-controlled crystal growth cell system. Crystal growth experiments for α-LGA have been conducted with 3 repeated runs under the same temperature and supersaturation conditions. A new mesh-fitting tool, CrystalSizer3D,^57^ was used to reconstruct 3D crystal structure from 2D images under transmission optical microscopy illumination. The tool builds and constructs a digital crystal mesh based on known α-LGA lattice parameters and crystal symmetry, with optical refractions calculated and the face-origin distances carefully tuned manually until the projected mesh matches the real crystals in the images.

The growth rates of all 20 habit faces of α-LGA were calculated from the fitted 3D crystals during their growth, with the relative supersaturations in every image being calculated from the crystal volume and physical data. A range of growth rate versus relative supersaturation data was collected and analyzed with respect to the 3 established growth rate kinetics models. Numerically, the results obtained span the relative supersaturation σ range of 0.65–0.78, and the measured growth rates are consistent with the analysis based upon the surface chemistry of the crystal facets. For example, the {110} and {100} faces have the highest growth rates (0.87 × 10^–8^ m/s and 0.98 × 10^–8^ m/s at σ = 0.78) and the {001} face has the lowest growth rate (0.22 × 10^–8^ m/s at σ = 0.78).

Direct observation of the growth process revealed that in α-LGA crystals, even the crystallographically equivalent faces tend to grow at quite different rates. Mechanistically, the growth of {111} and (001) faces was found to fit with the B&S model, while all other forms have better fits with the BCF model. Investigation of resistance parameters revealed that the growth rates of {111} and (001) faces were rate-limited by the MT process, while all other forms being limited by the SI process. Moreover, the MT process has a significant impact on growth, especially impacting on the differences in growth rates between top-facing and bottom-facing faces within the same form. This probably reflects the effect of the stagnant solution environment within the growth cell, which can cause different MT resistance to differently orientated habit faces. In future studies, this hypothesis might be validated by measuring the growth rates under a non-stagnant solution environment, such as an agitated or continuous flow growth cell.

GRD was observed between equivalent surfaces within the same morphological form and even on the same habit face between different crystals. This could be due to variability in the size and shape difference of the seed crystals, competition from neighboring faces, or slight differences in crystal perfection. The exact reason for GRD in these crystals remains unclear, but in future studies, further characterization of crystal defects could be conducted in order to evaluate the impact of defect content on the overall GRD behavior.

In future studies, it is also worth considering applying the methodology presented in this work to other crystal systems or larger-scale reactors. In the latter case, this could enable investigations of the variation of individual face-specific growth rates and the resulting crystal size distribution during representative batch crystallization processes. Development of a high-quality reactor-based imaging system in optical transmission mode together with more advanced image processing techniques may be required.

## Supplementary Material



## Notes

Initial and intermediate data from the experiments, including initial data of measured face-origin distances for all faces in 3 repeat runs; calculated volume and relative supersaturation of the crystal during its growth in 3 repeat runs; faceted growth rates vs relative supersaturation for each repeat run; list of averaged growth rates from measurements for all crystallographic forms under different relative supersaturations; list of parameters of faceted growth kinetics from the fitting of experimental growth rates with the kinetics models; and estimated topological structure of all forms of the crystal and growth rate dispersion on each habit face in different runs (PDF)

## §

School of Earth and Environment, University of Leeds, Leeds, LS2 9JT, UK

## ∥

School of Food Science and Nutrition, University of Leeds, Leeds, LS2 9JT, UK.

## Abbreviations

σ: relative supersaturation
σ_*t*_: relative supersaturation at time *t*
σ_crit_: critical relative supersaturation
*c*: concentration of solution
*c*_*e*_: equilibrium concentration (solubility)
*c*_0_: initial concentration of solution
*d*_*v*_: volume equivalent diameter
*r*_*v*_: volume equivalent radius
*m*_s_: initial mass of solute
ρ_s_: solute density
*V*_*t*_: volume of crystal at time *t*
*V*_0_: volume of crystal seed
*V*_*c*_: volume of cuvette
*G*: facet growth rate
$\frac{1}{k_{SI}}$: surface integration resistance
$\frac{1}{k_{MT}^{\prime }}$: mass transfer resistance
MW_s_: solute molecular weight
*k*_*G*_: growth rate constant
*r*: growth exponent
*A*_1_,*A*_2_: thermodynamic parameters
Adj.*R* ^2^: adjusted *R* ^2^
RSS: residual sum of squares
BCF: Burton–Cabrera–Frank model
B&S: Birth and Spread model
CCDC: the Cambridge Crystallographic Data Centre
GRD: growth rate dispersion
H-bond: hydrogen bond
LGA: l-Glutamic acid
MT: mass transfer
SI: surface integration
α-LGA: α-form l-glutamic acid
β-LGA: β-form l-glutamic acid
