# Supplementary material for: In Situ 3D Crystal Growth Measurement: Unravelling the Nature of Crystal Face-Specific Growth Rate Dispersion in α‑Form l‑Glutamic Acid
Source: Ind Eng Chem Res. 2026 Sep 30;65(40):21697–713. doi: 10.1021/acs.iecr.6c02035 (PMC13674084; doi:10.1021/acs.iecr.6c02035)
Supplement: Supplementary file 1 [file ie6c02035_si_001.pdf]

Supporting Information

In-situ 3D Crystal Growth Measurement: Unravelling the Nature of Crystal Face-specific Growth Rate Dispersion in α-form L-Glutamic Acid

*Chen Jiang [1], Thomas P. Ilett [2, 3], Thomas A. Hazlehurst [2, 4], Cai Y. Ma [1], David C. Hogg [2], Kevin J. Roberts [1, *]*

[1] School of Chemical and Process Engineering, University of Leeds, Leeds, LS2 9JT, UK

[2] School of Computer Science, University of Leeds, Leeds, LS2 9JT, UK

[3] Current address: School of Earth and Environment, University of Leeds, Leeds, LS2 9JT, UK

[4] Current address: School of Food Science and Nutrition, University of Leeds, Leeds, LS2 9JT, UK

[*] Corresponding author: k.j.roberts@leeds.ac.uk

This supplementary supports the main manuscript with:

S1. The initial data of measured face-origin distances for all faces in 3 repeat runs

S2. The calculated volume and relative supersaturation of the crystal during its growth in the 3 repeat runs, based on the measured face-origin distances on all the frames

S3. Facetted growth rates vs relative supersaturation for each repeat run

S4. Measured growth rates results for all crystallographic forms under different relative supersaturations

S5. Fitted parameters of experimental growth rates with the kinetics models

S6. The estimated topological structure of all forms of α-LGA crystal

S7. Growth rate dispersion on each habit face in different runs

S1. The initial data of measured face-origin distances for all faces in 3 repeat runs

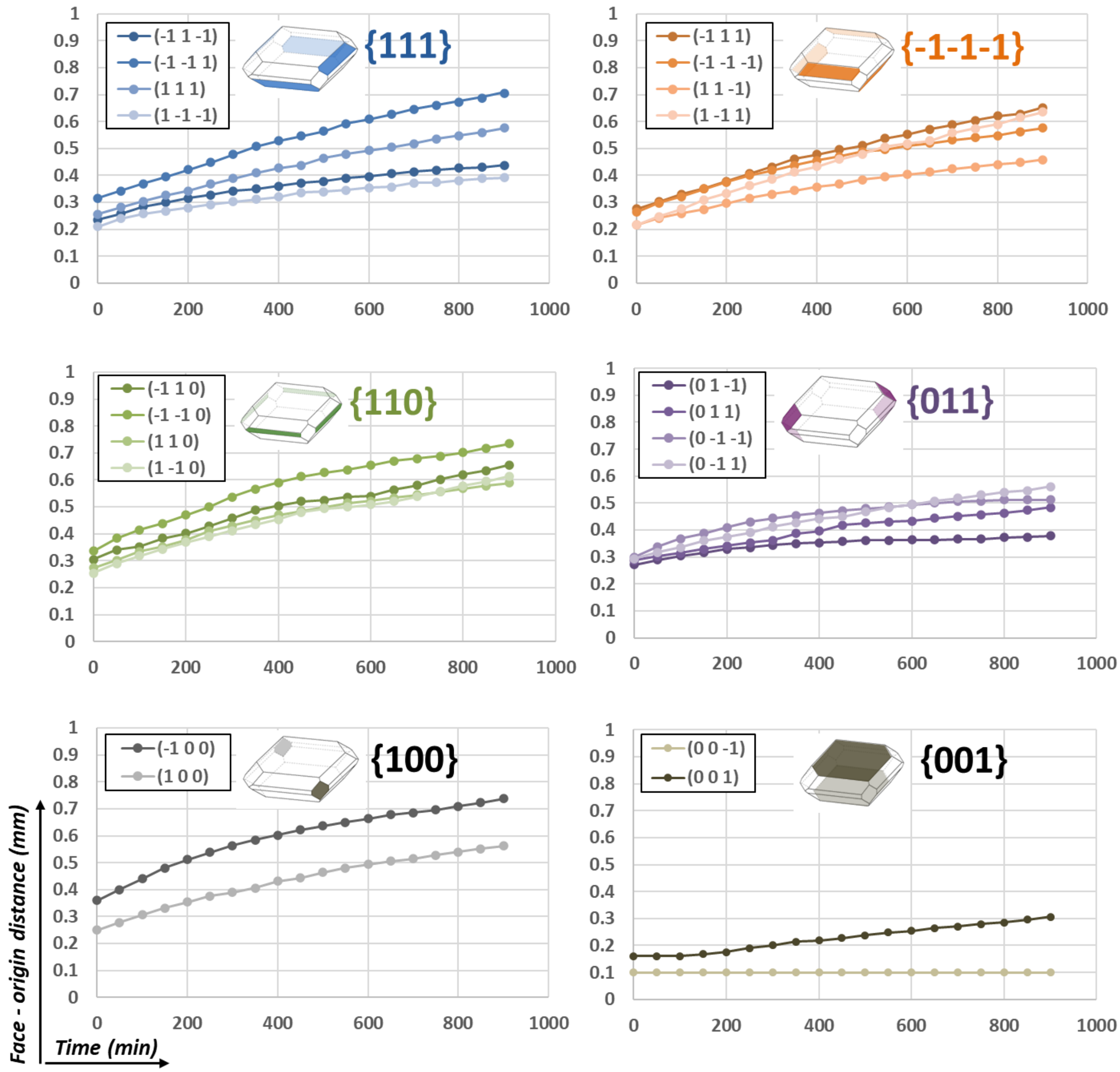

Figure S1. The initial data of measured face-origin distances for all faces in 3 repeat runs (run 1)

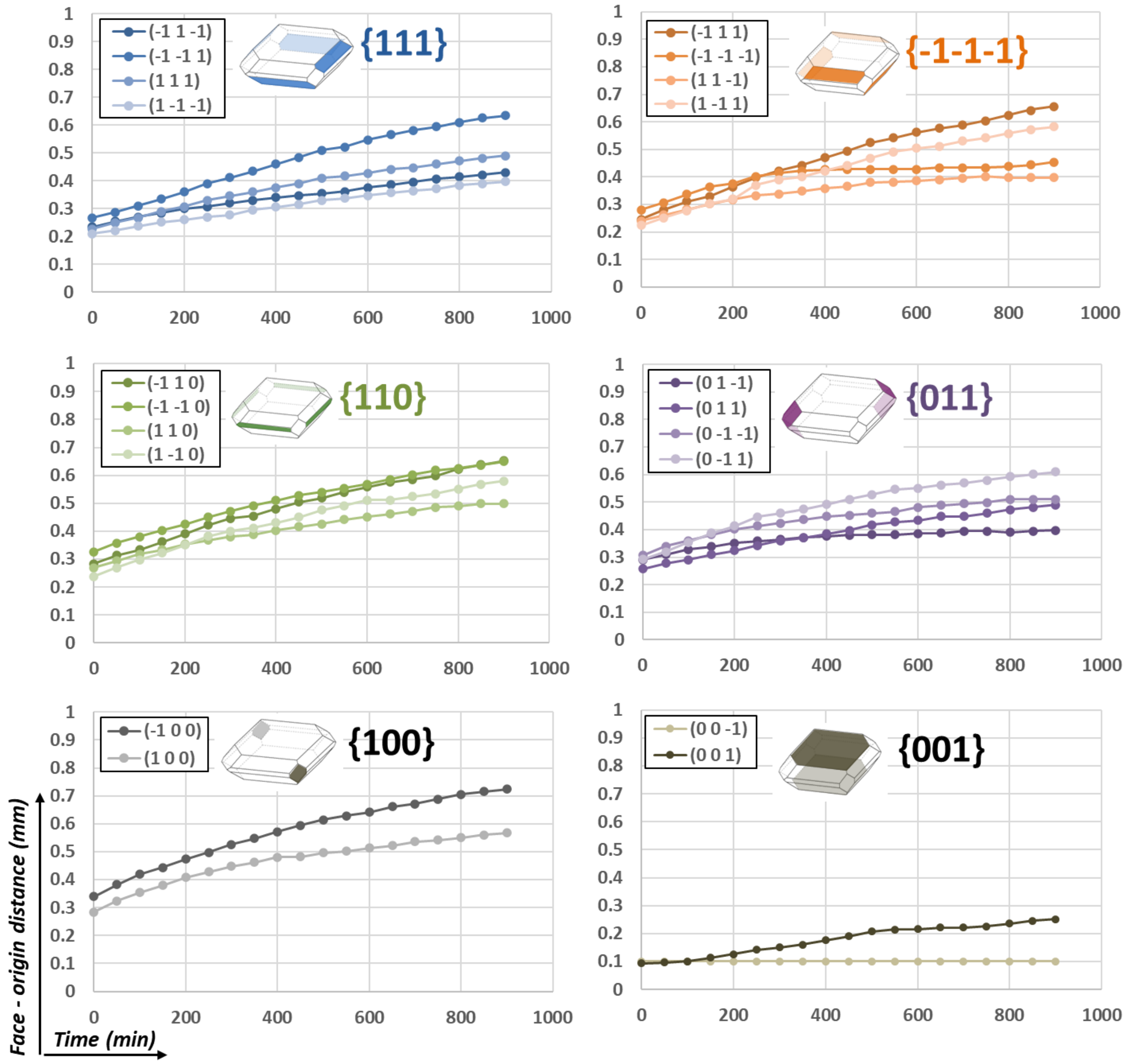

Figure S1. (continued) The initial data of measured face-origin distances for all faces in 3 repeat runs (run 2)

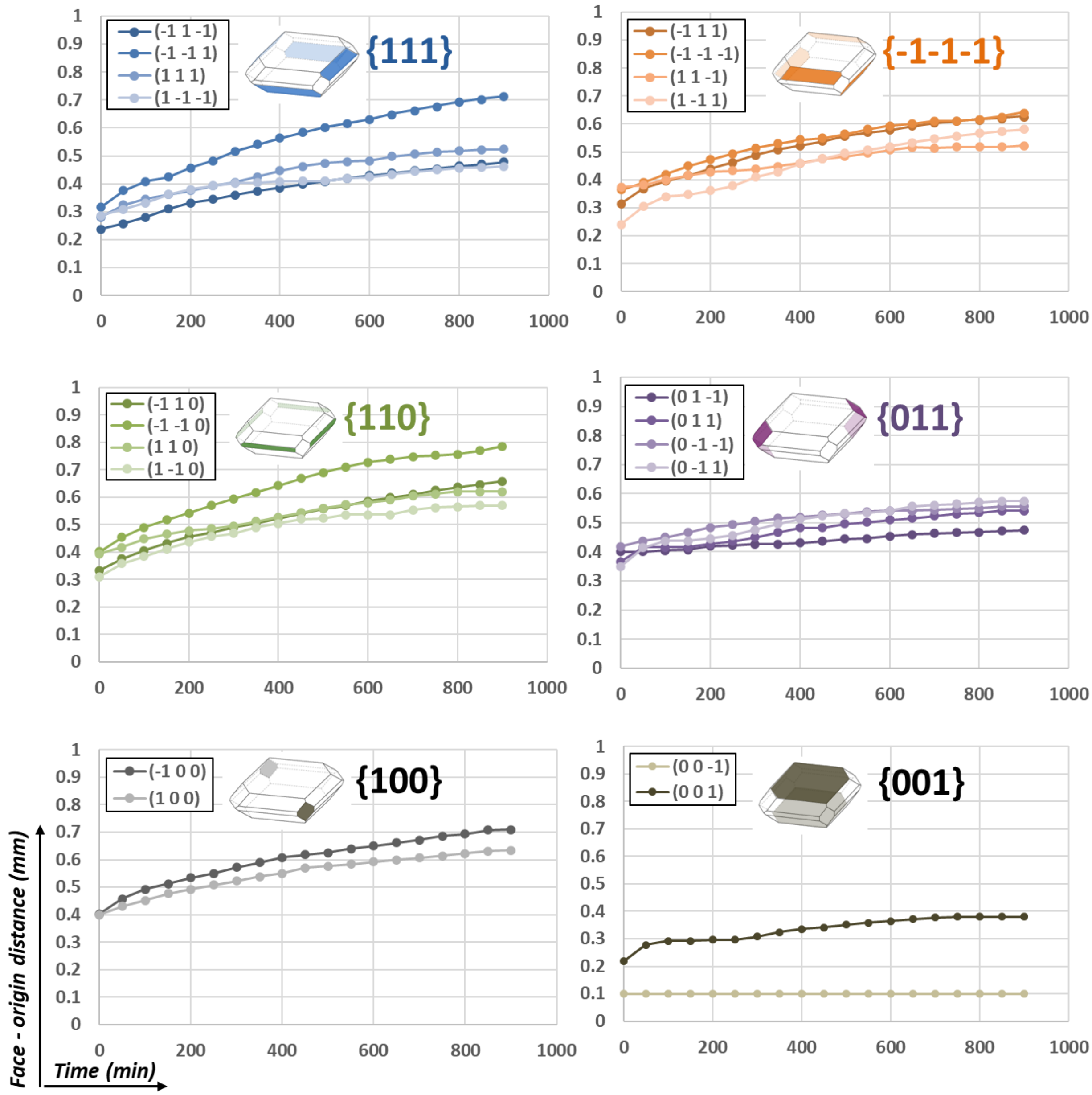

Figure S1. (continued) The initial data of measured face-origin distances for all faces in 3 repeat runs (run 3)

S2. The calculated volume and relative supersaturation of the crystal during its growth in 3 repeat runs, based on the measured face-origin distances on all the frames

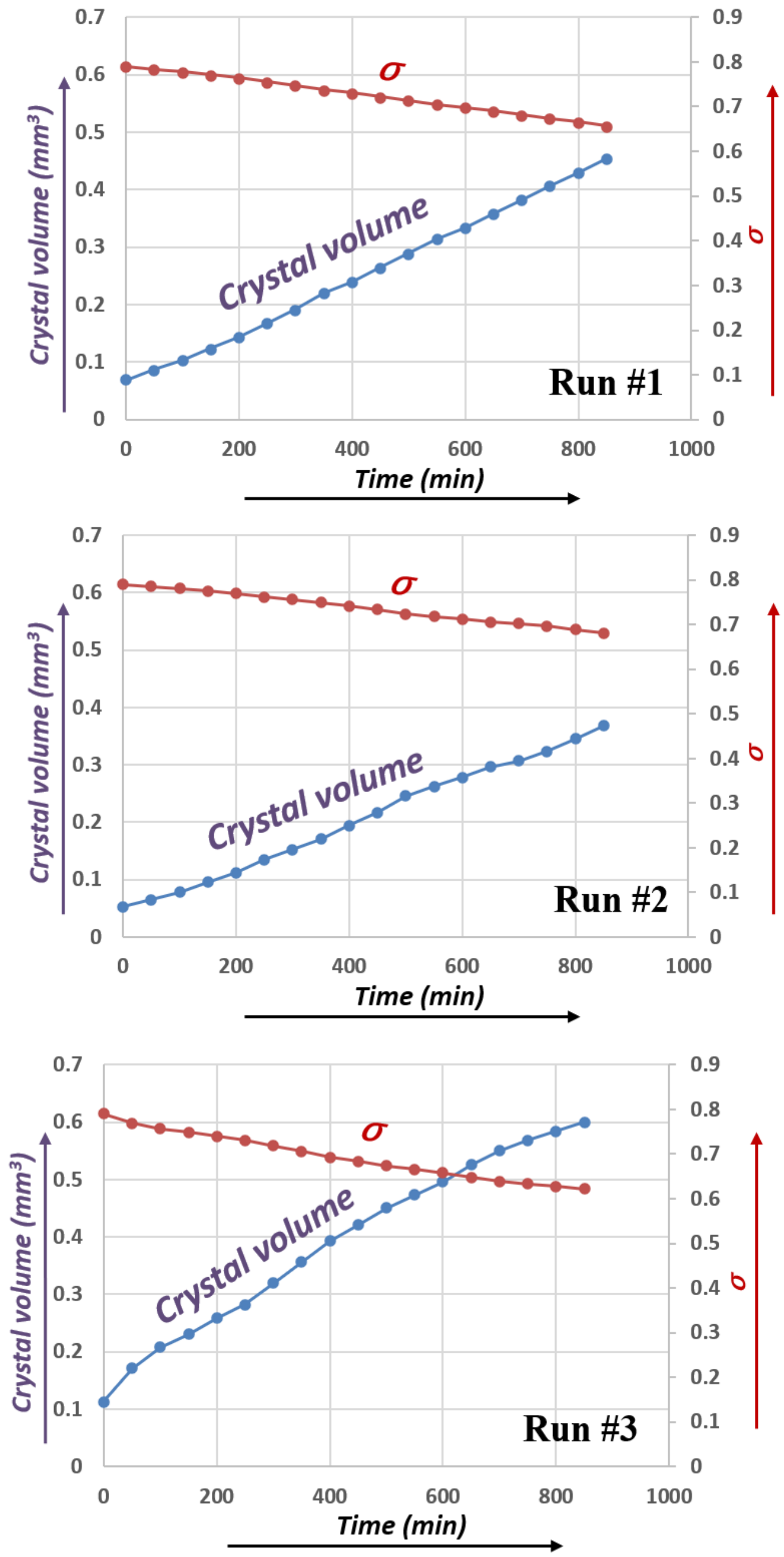

Figure S2. The calculated volume and relative supersaturation of the crystal during its growth in 3 repeat runs, based on the measured face-origin distances on all the frames

S3. Facetted growth rates vs relative supersaturation for each repeat run

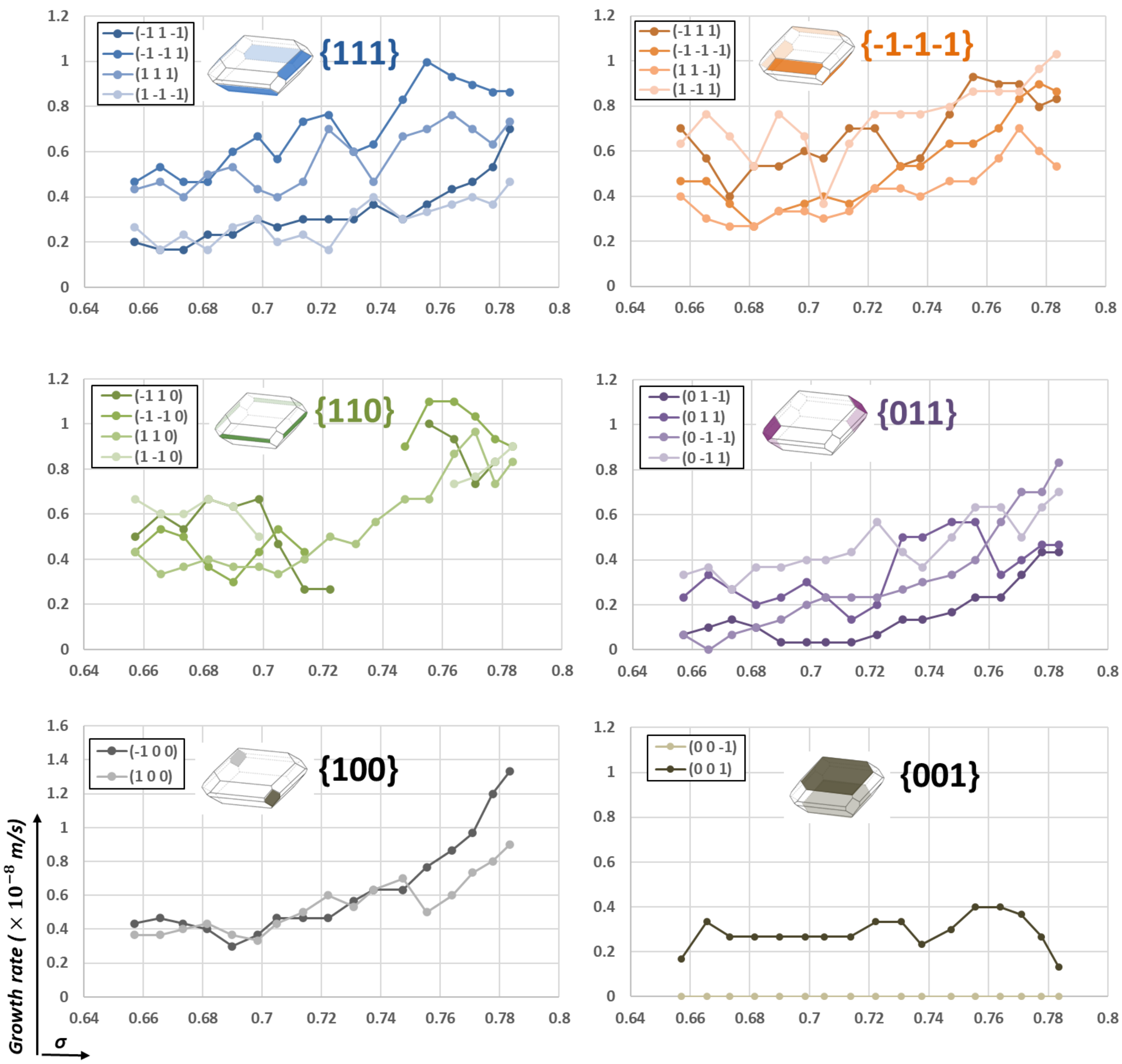

Figure S3. Facetted growth rates vs relative supersaturation for each repeat run, disappearing points indicate facet out (run 1)

Run #2

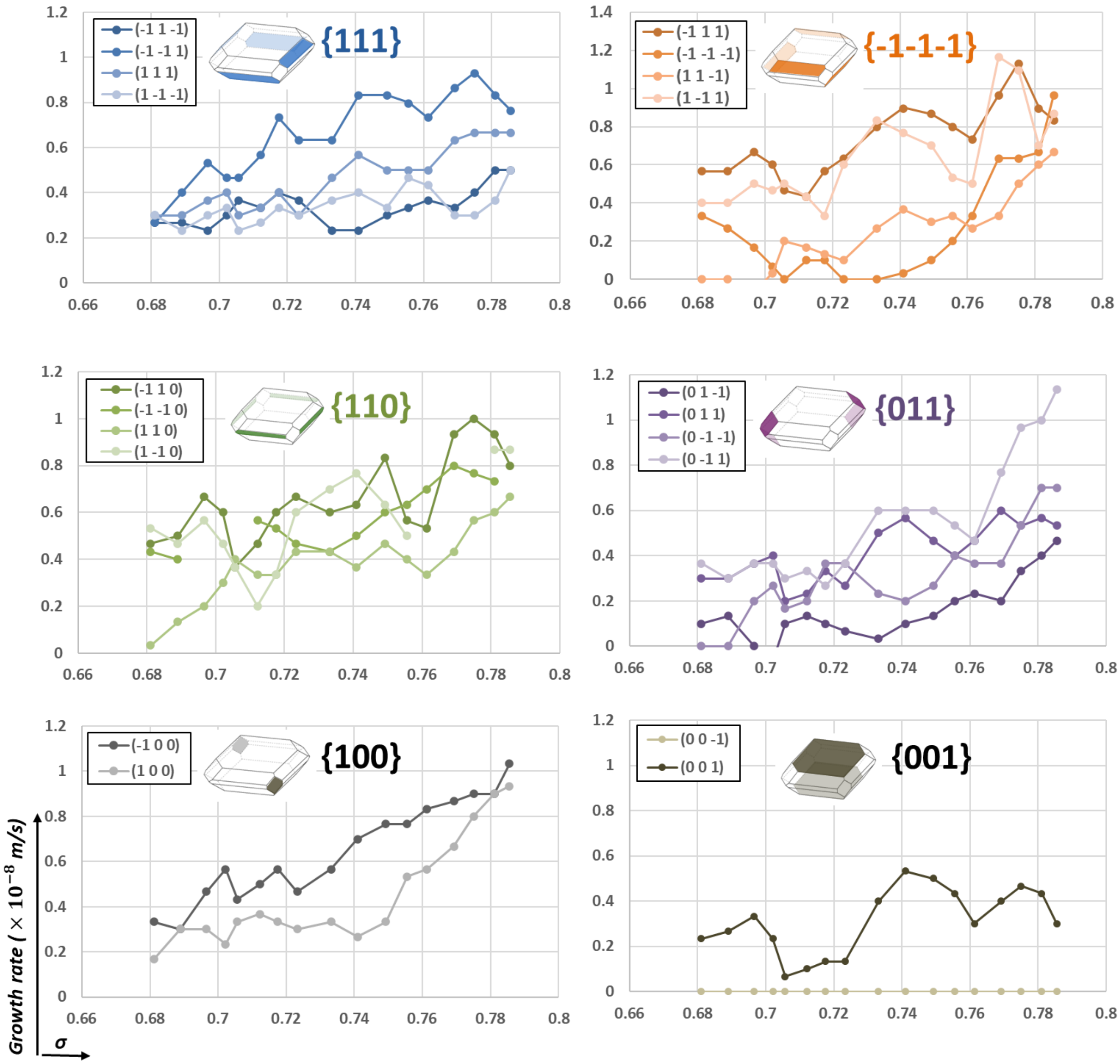

Figure S3. (continued) Facetted growth rates vs relative supersaturation for each repeat run, disappearing points indicate facet out (run 2)

Run #3

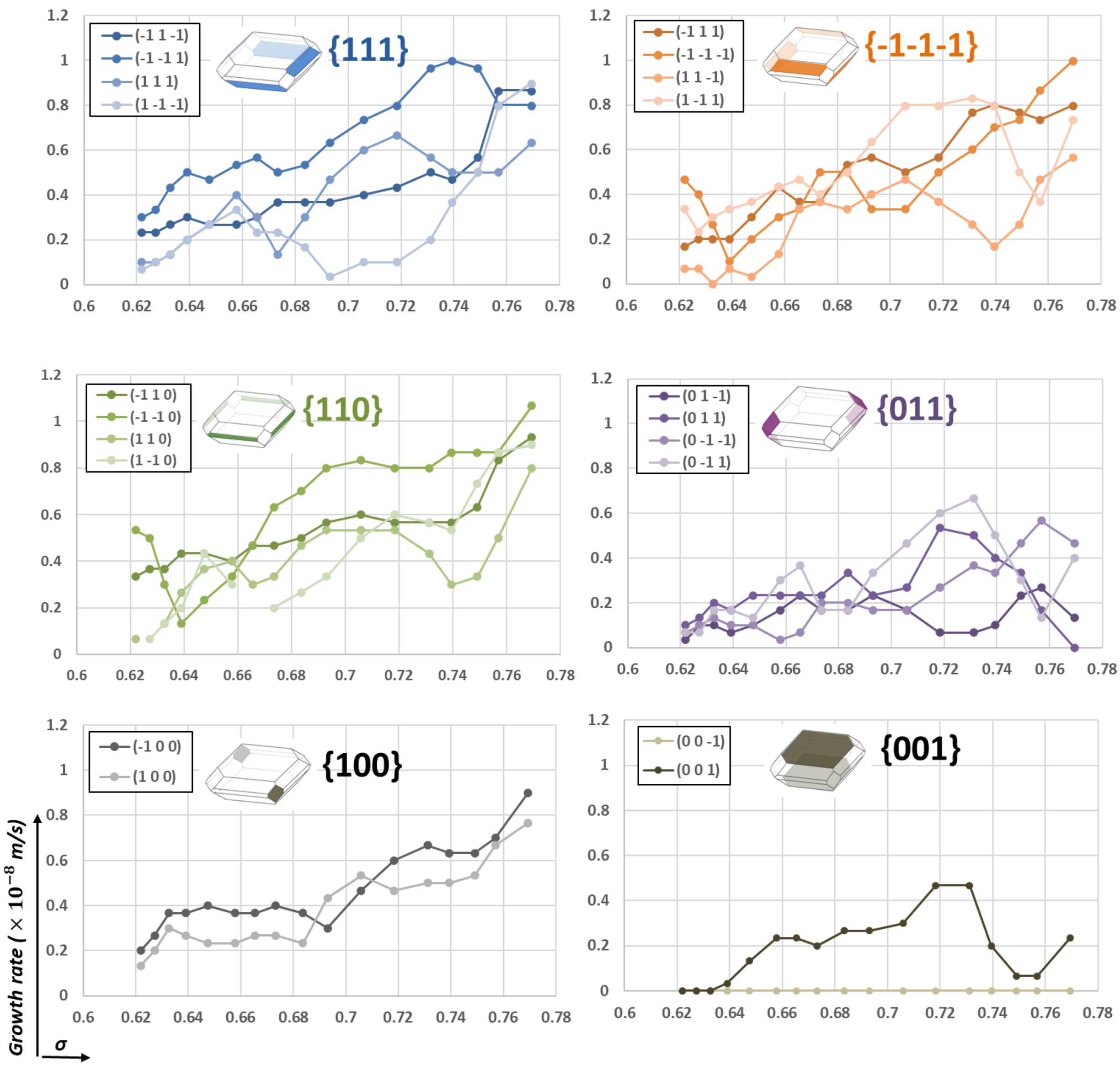

Figure S3. (continued) Facetted growth rates vs relative supersaturation for each repeat run, disappearing points indicate facet out (run 3)

S4. Measured growth rates results for all crystallographic forms under different relative supersaturations

Table S1. List of averaged growth rates from measurements for all crystallographic forms under different relative supersaturations

Form	{111}		{-1-1-1}		{110}	{011}		{100}	{001}	
Face	(111)<br>(-1-11)	(1-1-1)<br>(-11-1)	(1-11)<br>(-111)	(11-1)<br>(-1-1-1)	(110)<br>(-110)<br>(1-10)<br>(-1-10)	(011)<br>(0-11)	(01-1)<br>(0-1-1)	(100)<br>(-100)	(001)	r_v
orient	top facing	bottom facing	top facing	bottom facing	perpen-dicular	top facing	bottom facing	perpen-dicular	top facing	N/A
**σ**	**Growth rate ($\times$ 10^{-8} m/s)**									
0.779	0.744	0.656	0.850	0.767	0.867	0.539	0.506	0.978	0.222	0.576
0.772	0.717	0.572	0.744	0.683	0.794	0.494	0.511	0.861	0.256	0.531
0.765	0.778	0.439	0.878	0.611	0.764	0.506	0.433	0.761	0.300	0.529
0.758	0.783	0.378	0.917	0.517	0.733	0.539	0.300	0.689	0.333	0.519
0.749	0.744	0.367	0.772	0.428	0.670	0.550	0.278	0.639	0.389	0.502
0.740	0.711	0.322	0.711	0.417	0.617	0.522	0.239	0.617	0.400	0.480
0.731	0.628	0.317	0.700	0.356	0.619	0.444	0.194	0.561	0.344	0.431
0.722	0.617	0.278	0.694	0.350	0.552	0.444	0.183	0.467	0.378	0.418
0.713	0.567	0.267	0.689	0.328	0.487	0.394	0.156	0.428	0.333	0.371
0.703	0.461	0.300	0.556	0.278	0.445	0.267	0.178	0.400	0.200	0.286
0.696	0.494	0.289	0.444	0.267	0.397	0.306	0.172	0.406	0.211	0.291
0.689	0.489	0.300	0.500	0.233	0.414	0.300	0.128	0.361	0.200	0.284
0.681	0.439	0.272	0.489	0.183	0.412	0.244	0.106	0.344	0.156	0.245
0.674	0.422	0.256	0.444	0.133	0.409	0.278	0.106	0.378	0.178	0.248
0.668	0.389	0.222	0.456	0.178	0.397	0.272	0.106	0.378	0.200	0.252
0.661	0.356	0.194	0.456	0.250	0.375	0.250	0.072	0.317	0.200	0.244
0.653	0.311	0.222	0.467	0.289	0.369	0.233	0.056	0.272	0.133	0.219

S5. Fitted parameters of experimental growth rates with the kinetics models.

Table S2. List of fitted parameters of facetted growth kinetics model. The highlighted cells show the best fitting kinetics model for each form. The values in the brackets represent 95% confidence intervals for the corresponding system parameters.

		(111) (-1-11)	(1-1-1) (-11-1)	(-111) (1-11)	(11-1) (-1-1-1)	{110}
orientation		top facing	bottom facing	top facing	bottom facing	perpendicular
Power law	σ_{crit}	0.62 (±0.002)	0.47 (±0.005)	0.45 (±0.507)	0.55 (±0.047)	0.45 (±0.061)
	$\frac{1}{k'_{MT}}$	2.60×10^{2}	2.45×10^{2}	1.45×10^{7}	4.41×10^{6}	8.05×10^{4}
	$\frac{1}{k_{SI}}$	1.66×10^{7}	7.38×10^{7}	2.75×10^{7}	4.27×10^{7}	4.97×10^{7}
	r	0.61 (±0.006)	2.00 (±0.030)	2.00 (±3.688)	2.00 (±0.318)	2.00 (±0.346)
	R^2	0.95	0.77	0.87	0.87	0.96
	$Adj. R^2$	0.94	0.70	0.83	0.82	0.95
	RSS	1.96×10^{-18}	5.47×10^{-18}	5.76×10^{-18}	7.00×10^{-18}	1.76×10^{-18}
B&S	σ_{crit}	**0.59 (±0.002)**	**0.59 (±0.006)**	0.57 (±0.004)	0.65 (±0.004)	0.60 (±0.003)
	$\frac{1}{k'_{MT}}$	**2.16×10^{7}**	**3.64×10^{7}**	2.26×10^{7}	1.92×10^{7}	8.88×10^{-12}
	$\frac{1}{k_{SI}}$	**4.73×10^{2}**	**1.84×10^{-29}**	8.43×10^{1}	9.78×10^{-21}	2.15×10^{7}
	A_1	**3.42 (±0.048)**	**9.79 (±0.005)**	4.85 (±0.212)	3.73 (±0.031)	0
	R^2	**0.97**	**0.72**	0.87	0.74	0.93
	$Adj. R^2$	**0.96**	**0.63**	0.83	0.66	0.90
	RSS	**1.13×10^{-18}**	**6.69×10^{-18}**	5.58×10^{-18}	1.37×10^{-17}	3.27×10^{-18}
BCF	σ_{crit}	0.54 (±0.007)	0.58 (±0.021)	**0.55 (±0.017)**	**0.55 (±0.003)**	**0.48 (±0.002)**
	$\frac{1}{k'_{MT}}$	1.56×10^{-8}	2.69×10^{-7}	**7.19×10^{-9}**	**2.12×10^{-2}**	**1.55×10^{-2}**
	$\frac{1}{k_{SI}}$	3.10×10^{7}	2.65×10^{7}	**2.65×10^{7}**	**4.68×10^{7}**	**4.97×10^{7}**
	A_2	0.10 (±0.005)	0.01 (±0.002)	**0.01 (±0.001)**	**25.30 (±3.207)**	**24.87 (±4.908)**
	R^2	0.95	0.73	**0.89**	**0.88**	**0.96**
	$Adj. R^2$	0.93	0.64	**0.83**	**0.84**	**0.95**
	RSS	2.17×10^{-18}	6.40×10^{-18}	**5.82×10^{-18}**	**6.47×10^{-18}**	**6.95×10^{-20}**

		(011)<br>(0-11)	(01-1)<br>(0-1-1)	{100}	(001)	r_v
orientation		top facing	bottom facing	perpendicular	top facing	N/A
Power law	σ_{crit}	0.59 (±0.087)	0.60 (±0.078)	0.51 (±0.049)	0.65 (±0.007)	**0.51 (±0.028)**
	$\frac{1}{k'_{MT}}$	6.14×10^{4}	3.98×10^{-7}	2.26×10^{6}	3.03×10^{4}	**1.58×10^{-8}**
	$\frac{1}{k_{SI}}$	3.34×10^{7}	5.86×10^{7}	3.88×10^{7}	2.46×10^{7}	**5.61×10^{7}**
	r	0.94 (±0.678)	2.00 (±1.295)	2.00 (±0.283)	0.29 (±0.030)	**1.70 (±0.147)**
	R^2	0.89	0.92	0.93	0.48	**0.97**
	$Adj. R^2$	0.85	0.89	0.91	0.30	**0.96**
	RSS	2.74×10^{-18}	2.73×10^{-18}	4.59×10^{-18}	6.34×10^{-18}	**8.21×10^{-19}**
B&S	σ_{crit}	0.60 (±0.014)	0.65 (±0.009)	0.61 (±0.003)	**0.62 (±0.012)**	N/A
	$\frac{1}{k'_{MT}}$	2.93×10^{7}	2.98×10^{7}	1.98×10^{7}	**3.23×10^{7}**	
	$\frac{1}{k_{SI}}$	4.19×10^{3}	7.42×10^{-13}	5.52×10^{-45}	**3.08×10^{4}**	
	A_1	2.35 (±0.101)	0	11.99 (±2.738)	**1.88 (±0.947)**	
	R^2	0.90	0.85	0.89	**0.83**	
	$Adj. R^2$	0.87	0.80	0.85	**0.77**	
	RSS	2.33×10^{-18}	5.07×10^{-18}	7.52×10^{-18}	**2.11×10^{-18}**	
BCF	σ_{crit}	**0.44 (±0.031)**	**0.60 (±0.014)**	**0.51 (±0.003)**	0.53 (±0.026)	N/A
	$\frac{1}{k'_{MT}}$	**1.06×10^{-5}**	**5.23×10^{-1}**	**4.08×10^{-2}**	6.14×10^{7}	
	$\frac{1}{k_{SI}}$	**7.22×10^{7}**	**5.87×10^{7}**	**4.07×10^{7}**	1.03×10^{7}	
	A_2	**0.93 (±0.835)**	**4.18 (±1.058) $\times10^{2}$**	**6.12 (±1.070)**	0.23 (±0.764)	
	R^2	**0.88**	**0.92**	**0.94**	0.42	
	$Adj. R^2$	**0.84**	**0.89**	**0.91**	0.22	
	RSS	**2.91×10^{-18}**	**2.73×10^{-18}**	**4.30×10^{-18}**	7.06×10^{-18}	

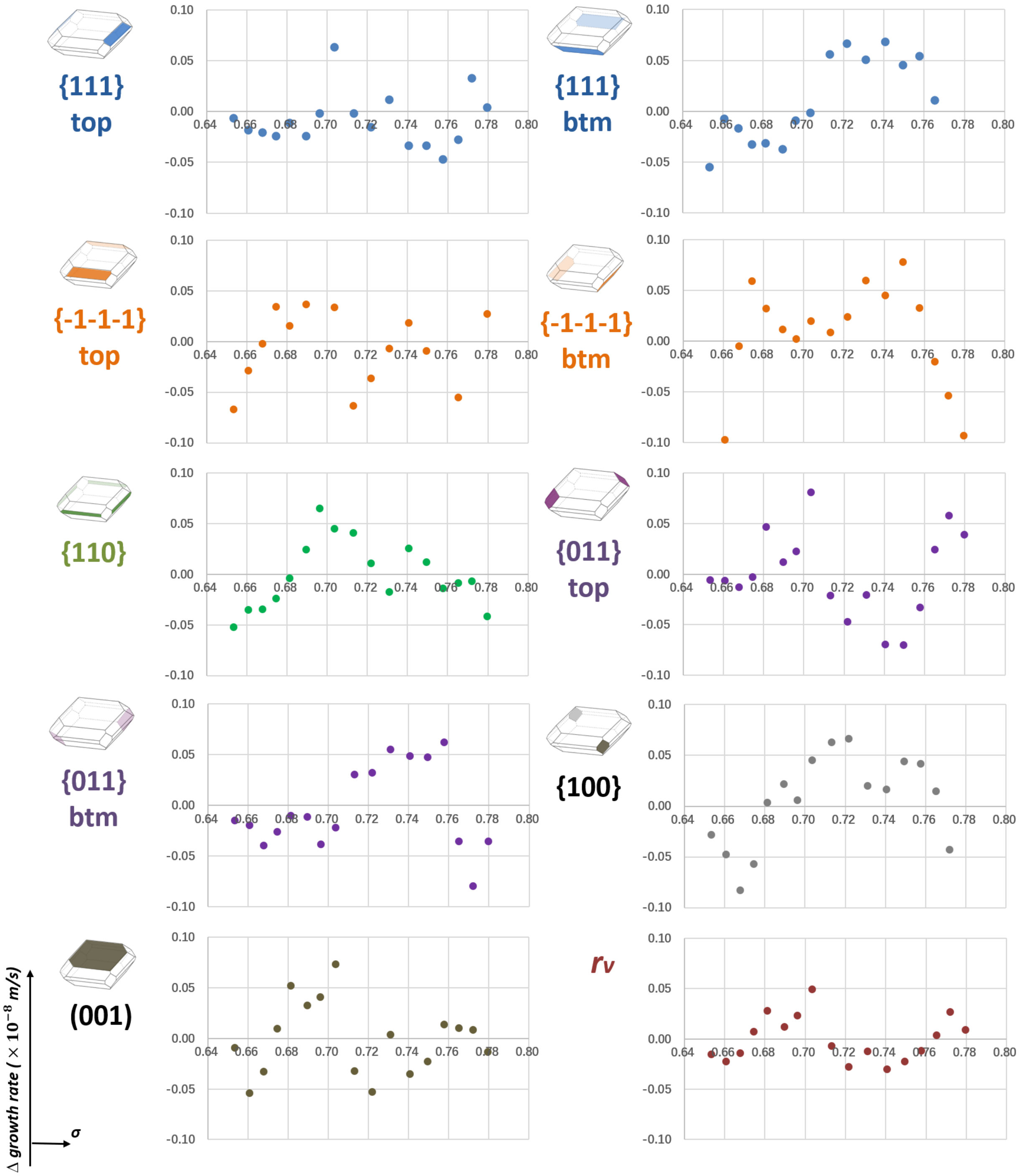

Figure S4. The residual plots of the best fitted growth kinetics model for each crystallographic form.

S6. The estimated topological structure of all forms of the crystal

Table S3. Estimated topological structure of all crystallographic forms of the crystal

Form	Surface topological structure	Rugosity
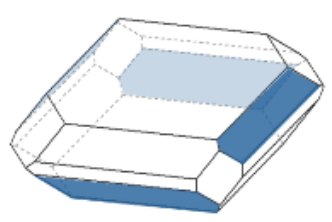 {111}	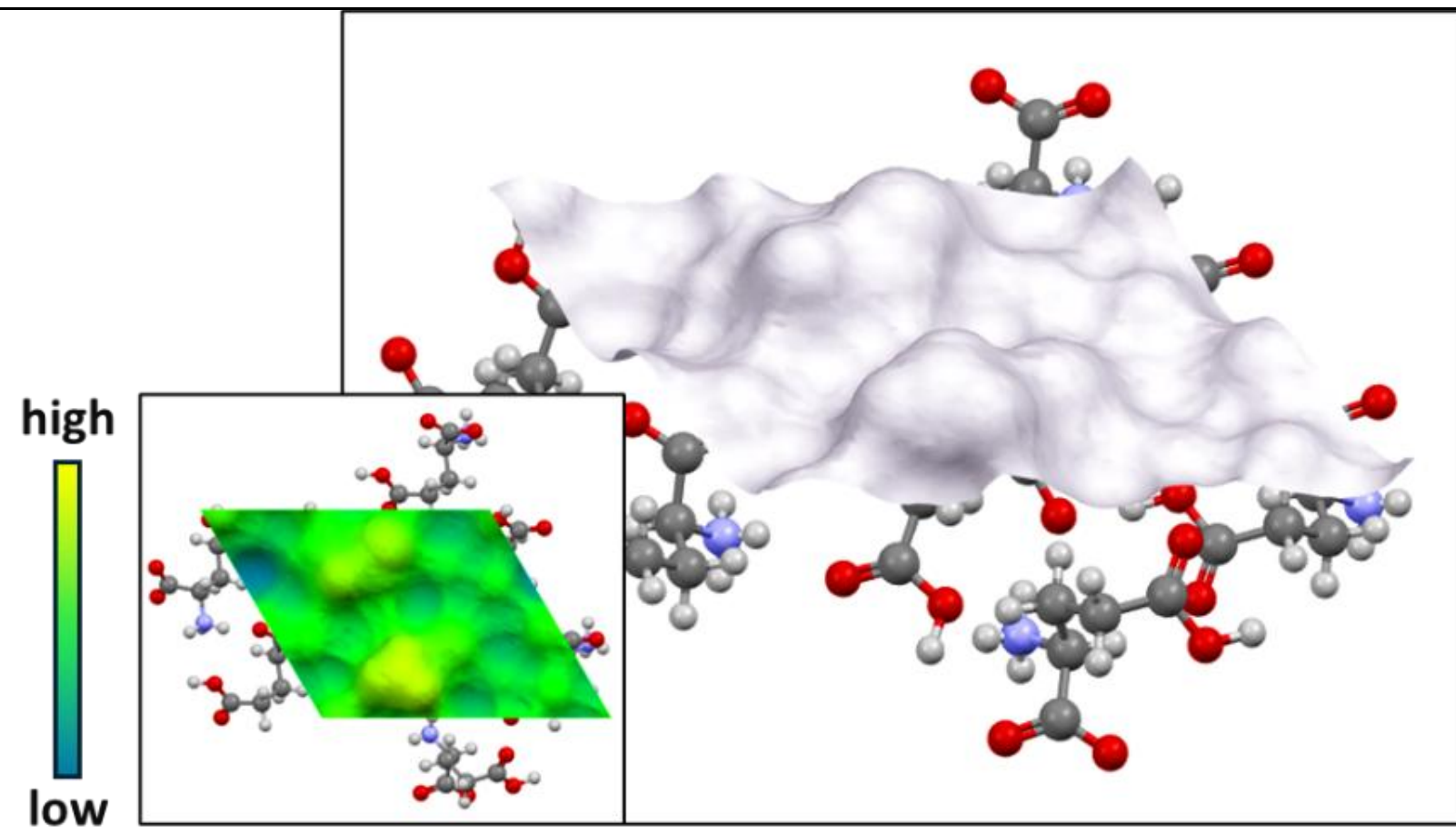	1.281
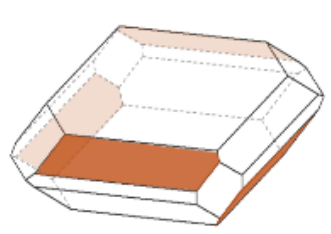 {-1-1-1}	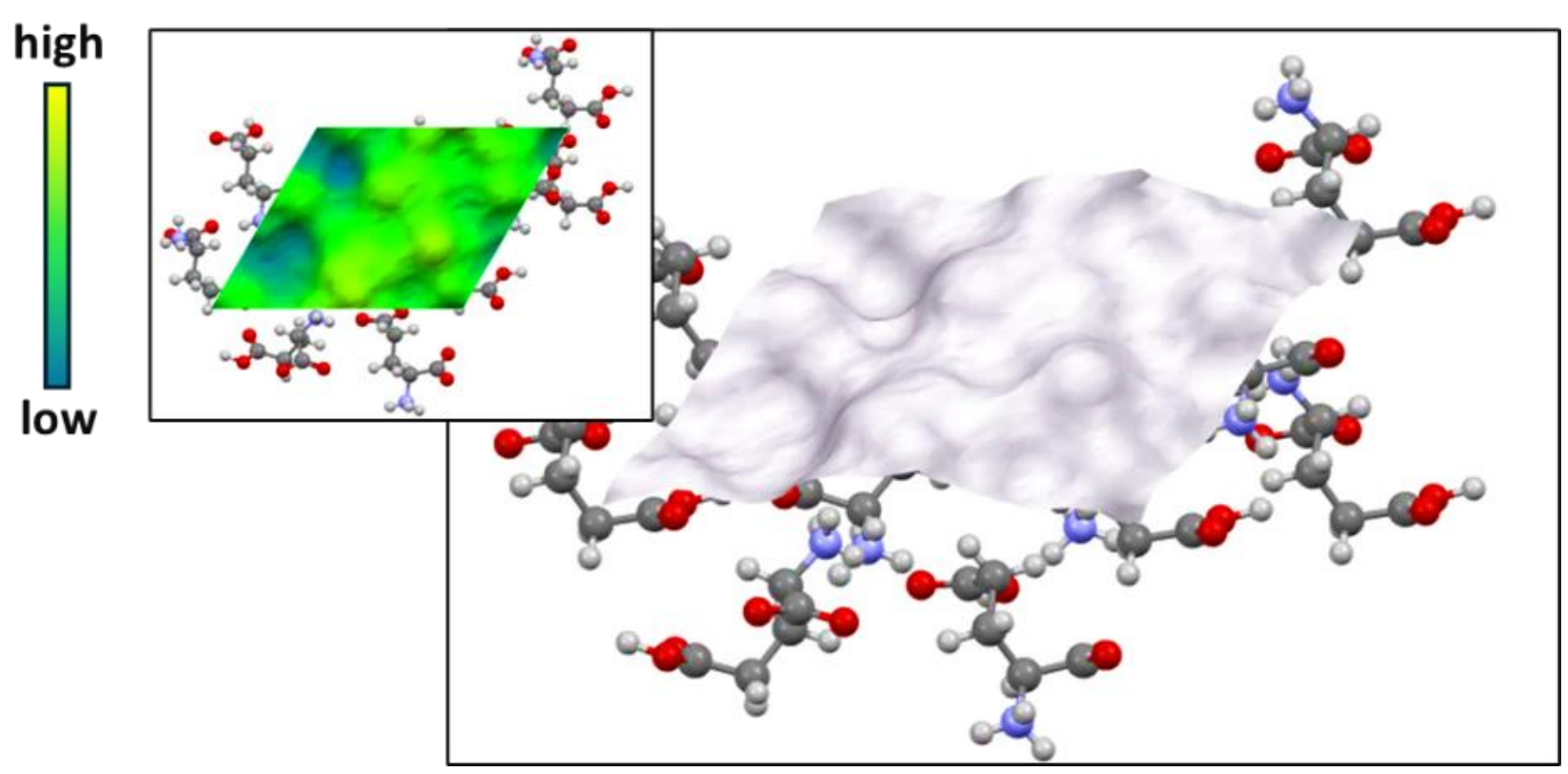	1.234
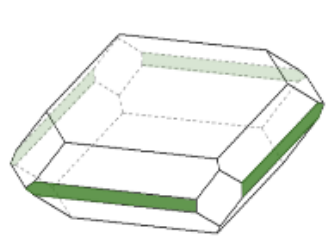 {110}	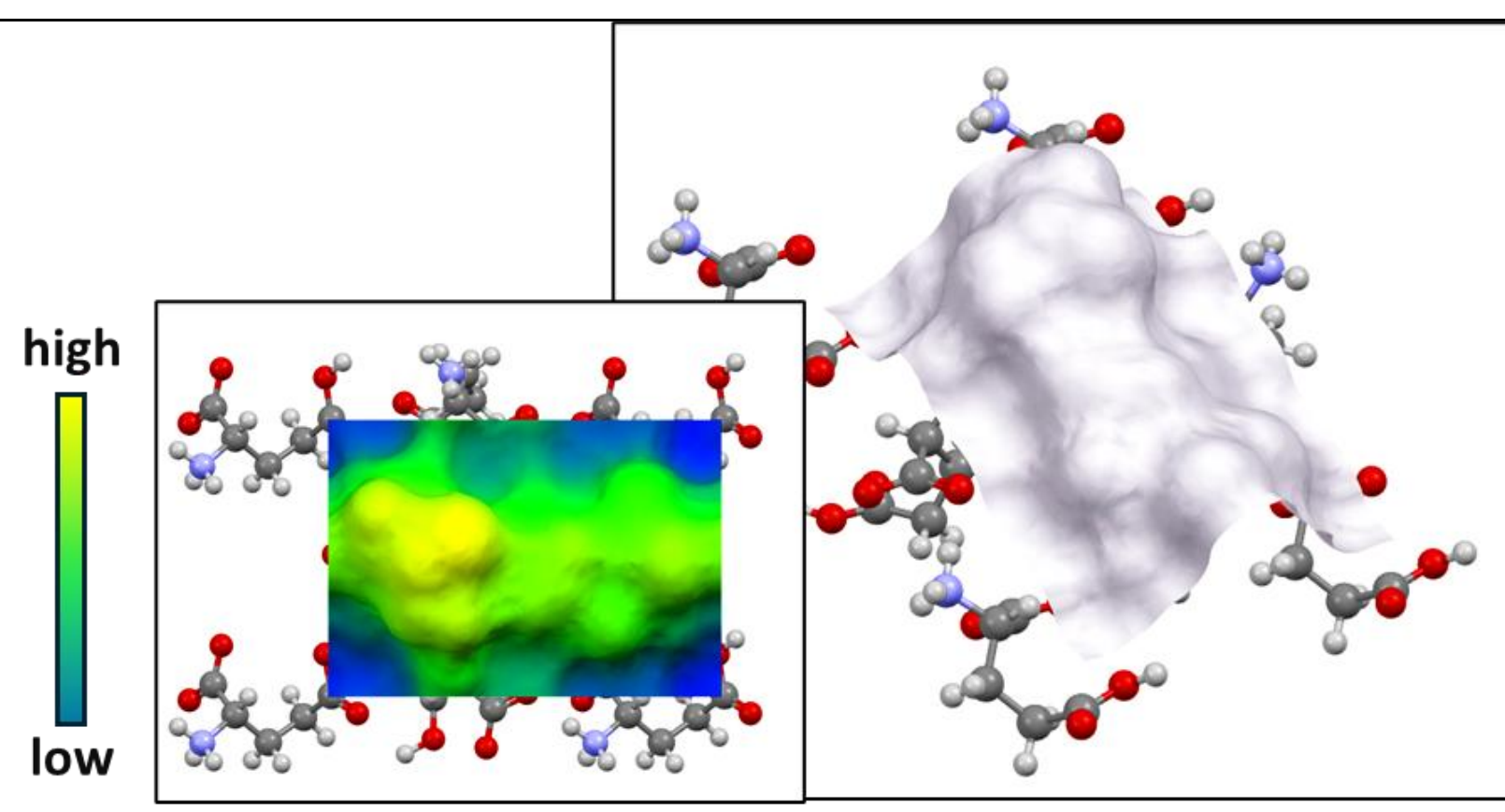	1.504

Form	Surface topological structure	Rugosity
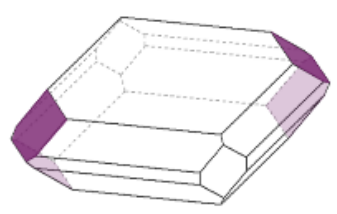 {011}	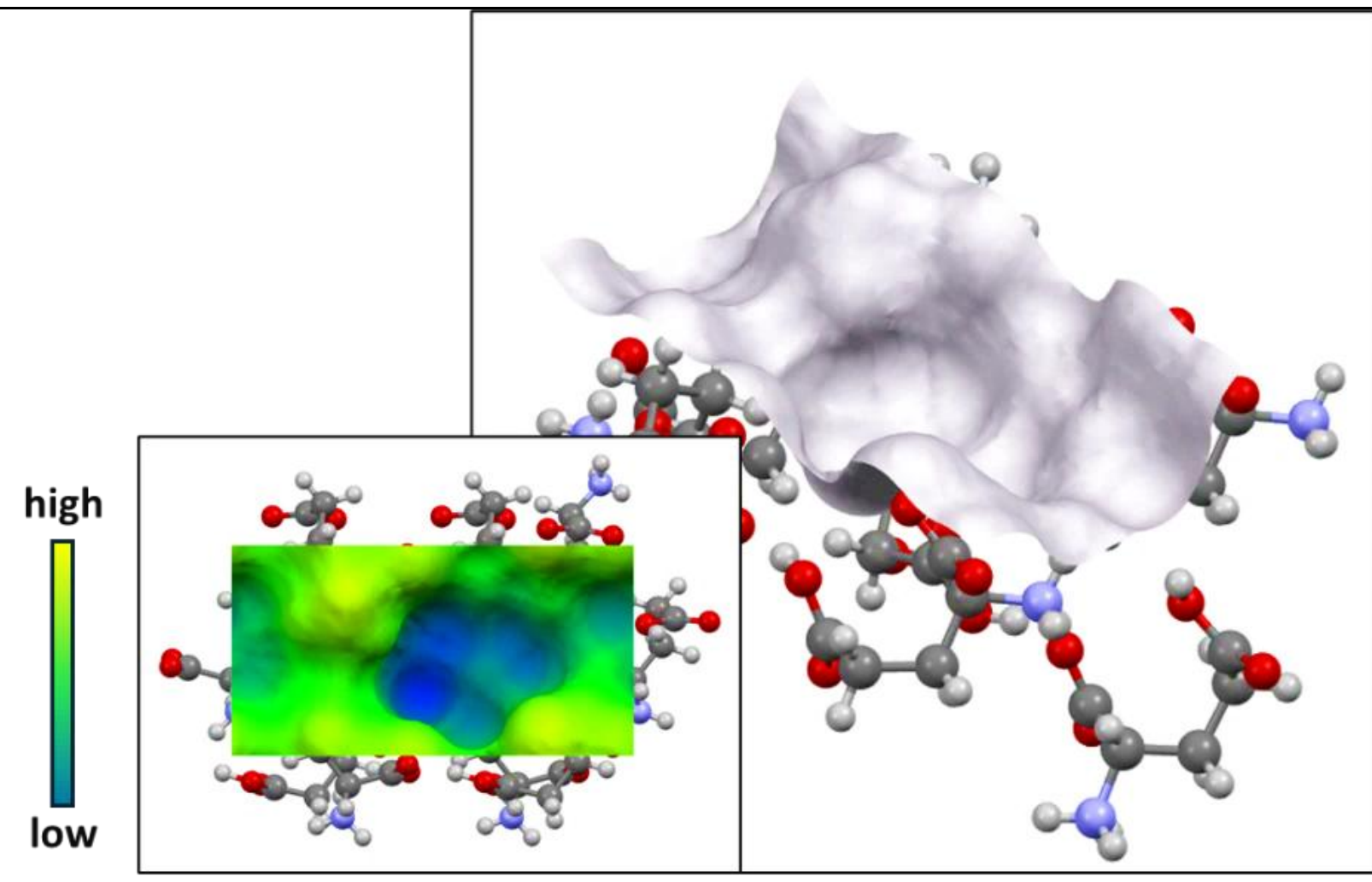	1.516
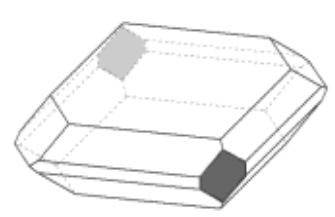 {100}	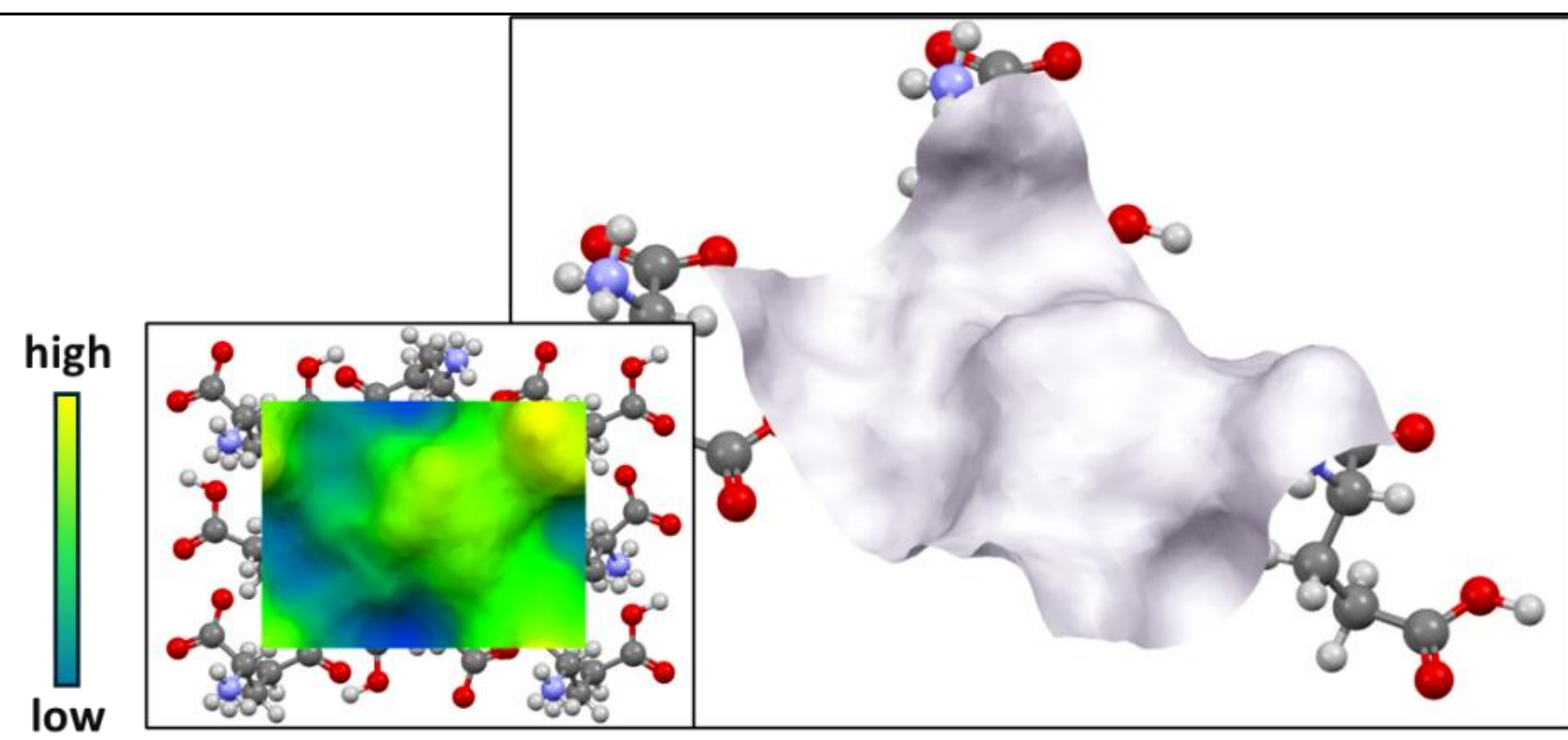	1.429
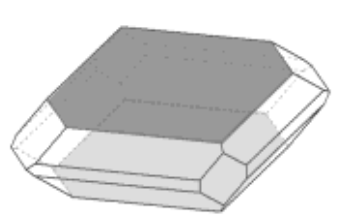 {001}	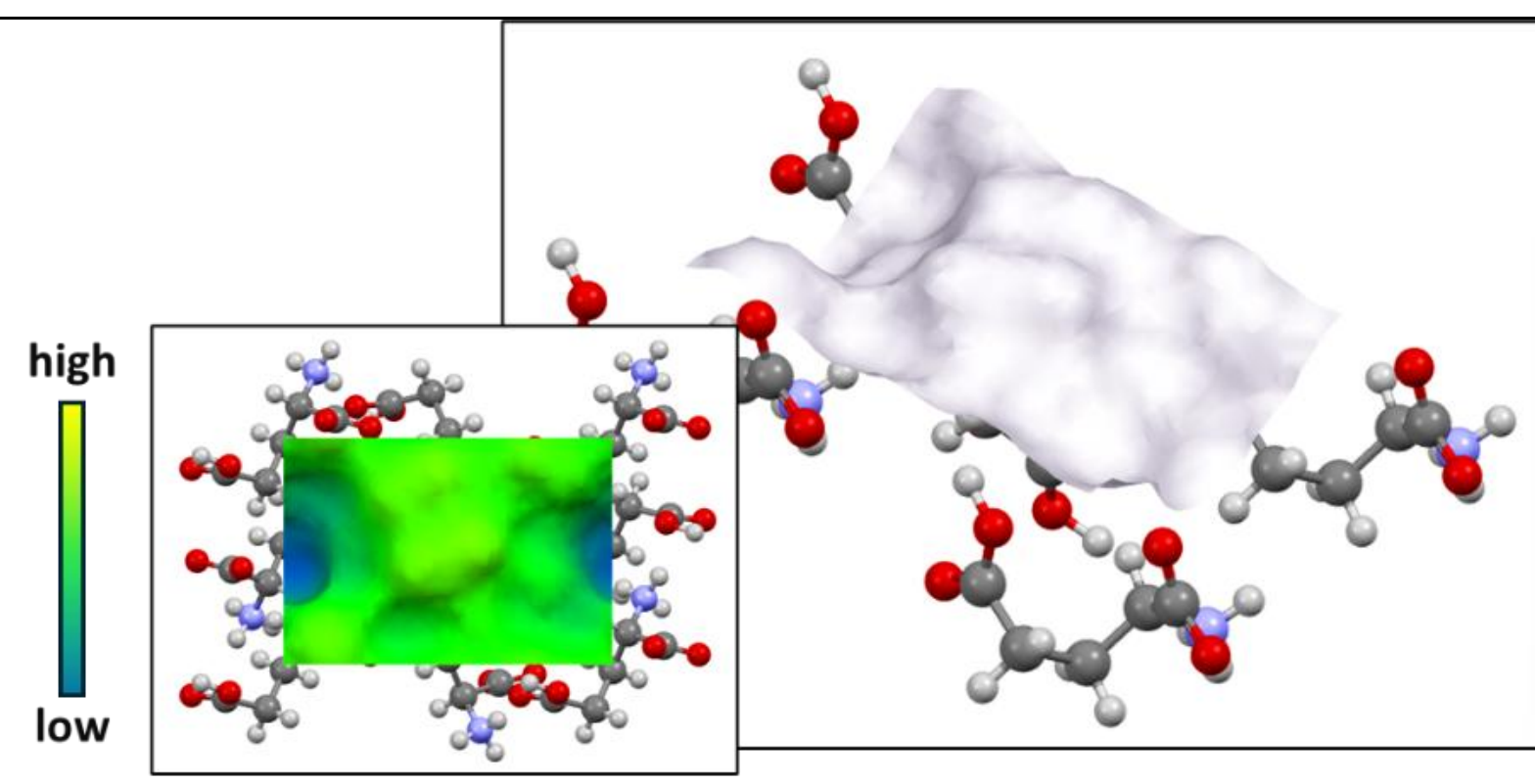	1.252

S7. Growth rate dispersion on each habit face in different runs.

Disappearing points indicate facet out.

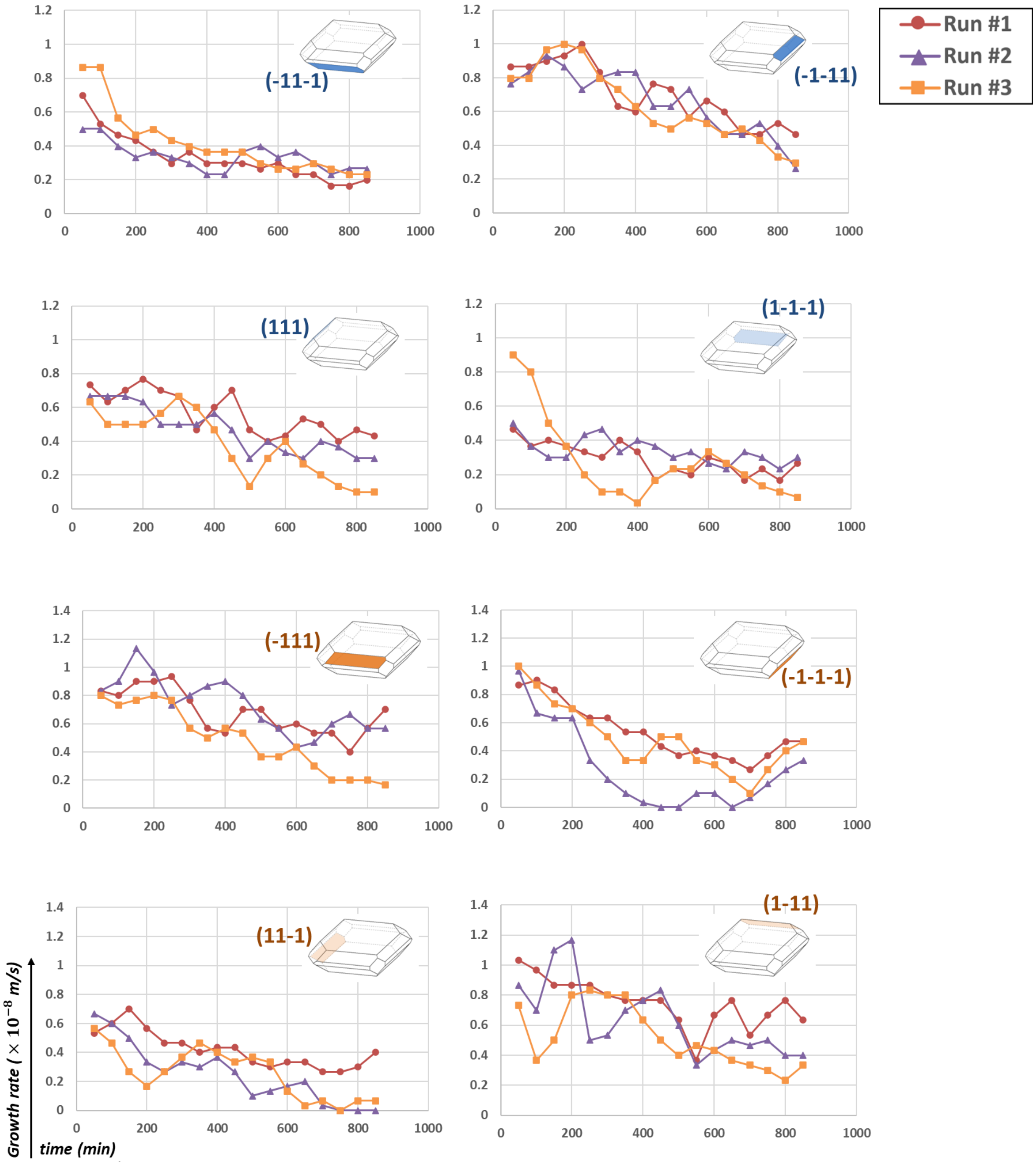

Figure S5. Growth rate dispersion on each habit face in different forms. Disappearing points indicate facet out.

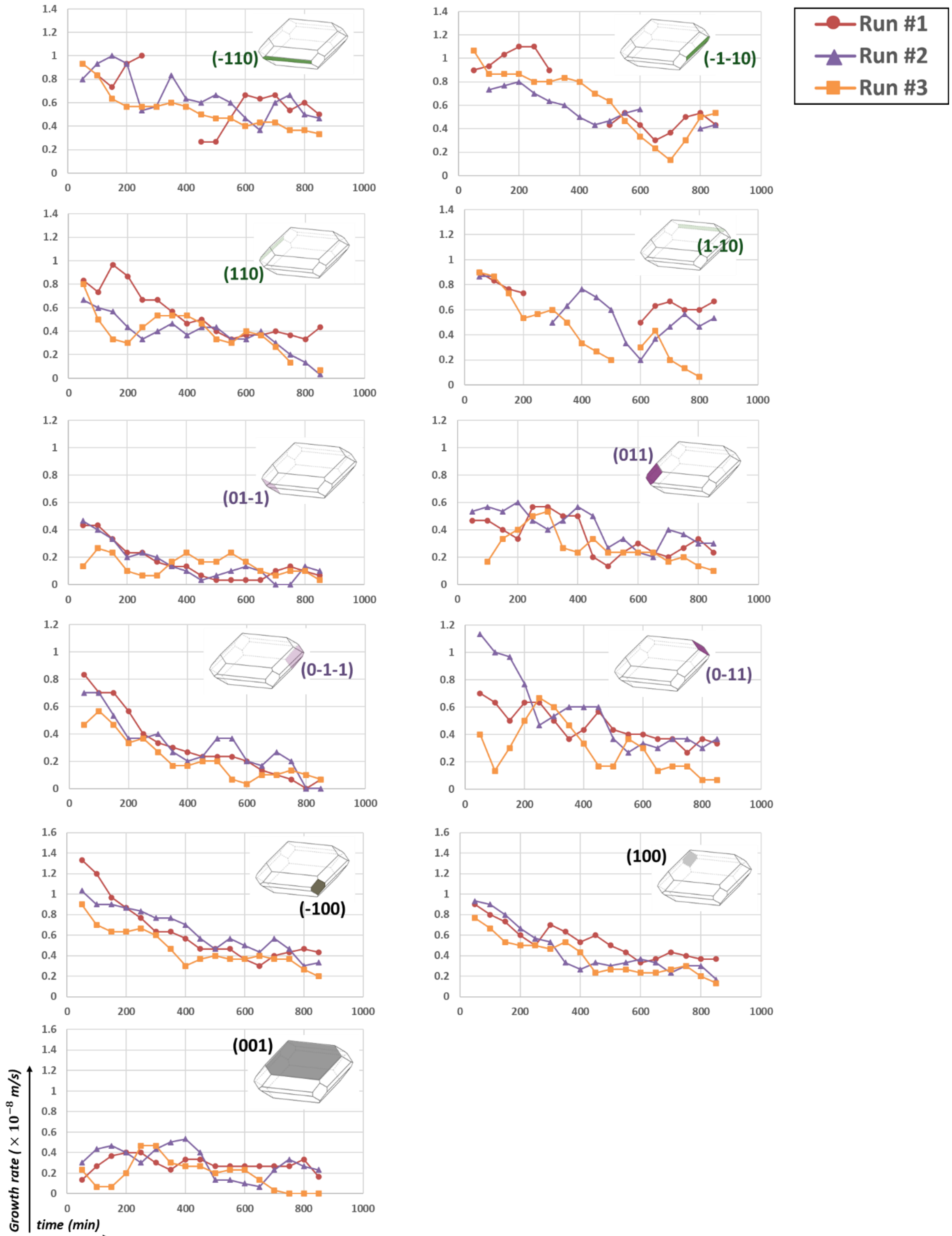

Figure S5. (continued) Growth rate dispersion on each habit face in different forms. Disappearing points indicate facet out.
